# Müller Glia maintain their regenerative potential despite degeneration in the aged zebrafish retina

**DOI:** 10.1111/acel.13597

**Published:** 2022-03-22

**Authors:** Raquel R. Martins, Mazen Zamzam, Dhani Tracey‐White, Mariya Moosajee, Ryan Thummel, Catarina M. Henriques, Ryan B. MacDonald

**Affiliations:** ^1^ The Bateson Centre Healthy Lifespan Institute MRC‐Arthritis Research UK Centre for Integrated research into Musculoskeletal Ageing and Department of Oncology and Metabolism University of Sheffield Medical School Sheffield UK; ^2^ 12267 Department of Ophthalmology, Visual and Anatomical Sciences Wayne State University School of Medicine Detroit Michigan USA; ^3^ Institute of Ophthalmology University College London London UK; ^4^ 376570 Moorfields Eye Hospital NHS Foundation Trust London UK; ^5^ 376570 Great Ormond Street Hospital for Children NHS Foundation Trust London UK; ^6^ 376570 The Francis Crick Institute London UK

**Keywords:** ageing, degeneration, Müller glia, proliferation, regeneration, retina, telomerase, Zebrafish

## Abstract

Ageing is a significant risk factor for degeneration of the retina. Müller glia cells (MG) are key for neuronal regeneration, so harnessing the regenerative capacity of MG in the retina offers great promise for the treatment of age‐associated blinding conditions. Yet, the impact of ageing on MG regenerative capacity is unclear. Here, we show that the zebrafish retina undergoes telomerase‐independent, age‐related neurodegeneration but that this is insufficient to stimulate MG proliferation and regeneration. Instead, age‐related neurodegeneration is accompanied by MG morphological aberrations and loss of vision. Mechanistically, yes‐associated protein (Yap), part of the Hippo signalling, has been shown to be critical for the regenerative response in the damaged retina, and we show that Yap expression levels decline with ageing. Despite this, morphologically and molecularly altered aged MG retain the capacity to regenerate neurons after acute light damage, therefore, highlighting key differences in the MG response to high‐intensity acute damage *versus* chronic neuronal loss in the zebrafish retina.

## Graphical abstract

Our data show that aged, molecularly and morphologically altered MG can still be stimulated to regenerate in response to an acute damage, highlighting key differences in MG response when compared to the chronic damage in ageing.

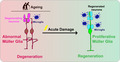

**Abbreviations**
ACs: amacrine cells
ANOVA: analysis of variance
BCA: biochonic acid
BCs: bipolar cells
BrdU: bromodeoxyuridine
BSA: bovine serum albumin
CMZ: ciliary marginal zone
Cralbp: retinaldehyde‐binding protein
DAPI: 4′,6‐diamidino‐2‐phenylindole
DMSO: dimethyl sulfoxide
DNA: deoxyribonucleic acid
DPX: dibutylphthalate polystyrene xylene
EDTA: ethylenediaminetetraacetic acid
EdU: 5‐ethynyl‐2′‐deoxyuridine
GABA: gamma‐aminobutyric acid
GCL: ganglion cell layer
Gfap: glial fibrillary acidic protein
GS: glutamine synthetase
HCs: horizontal cells
IMS: industrial methylated spirit
INL: inner nuclear layer
IPL: inner plexiform layer
LDS: lithium dodecyl sulphate
MES: 2‐(N‐morpholino) ethanesulfonic acid
MG: müller glia cells
MS‐222: ethyl 3‐aminobenzoate methanesulfonate
OKR: optokinetic response
ON: overnight
ONL: outer nuclear layer
ONL: outer nuclear layer
OPL: outer plexiform layer
PBS: phosphate‐buffered saline
PCNA: proliferating cell nuclear antigen
PFA: paraformaldehyde
PRL: photoreceptor layer
PVDF: polyvinylidene difluoride
RGCs: retinal ganglion cells
RIPA: radioimmunoprecipitation assay
RPE: retinal pigment epithelium
RT: room temperature
SDS: sodium dodecyl sulphate
SEM: standard error of mean
TBS: TRIS buffered saline
*tert* ^−^^/^^−^
: telomerase mutant hu3430
TNF: tumour necrosis factor
WT: wild type
Yap: yes‐associated protein
ZO1: zonula occludens‐1

## 1 INTRODUCTION

The physiology and structure of the healthy human eye is known to degrade with age. Hallmarks of the ageing human retina include tissue thinning, neuronal loss, especially in the macula, and reduced visual function (Arden & Jacobson, 1978; Marshall, 1987; Salvi et al., 2006; Skalka, 1980; Slataper, 1950; Weale, 1975). Accordingly, ageing is a significant risk factor for degeneration and disease in the retina, such as age‐related macular degeneration or primary open angle glaucoma (Al‐Ubaidi et al., 2013; Marshall, 1987). Retinal degeneration is often a process that plays out over months or years, whereby neurons gradually die leading to dysfunction (Marshall, 1987; Salvi et al., 2006). Regenerative‐based therapies, such as stimulating endogenous Müller Glia (MG) to regenerate dying neurons, offer a great promise for the treatment of various types of degenerative blinding conditions, including those associated with ageing. Thus, determining the effects of ageing on the regenerative potential of the retina is an important consideration for the efficacy of such potential treatments going forward.

Müller Glia are the principal cells tasked with supporting and maintaining the retina throughout life. They provide retinal neurons with a myriad of supportive functions, including trophic support, neurotransmitter recycling and energy metabolism (Reichenbach & Bringmann, 2013). While these functions are critical for healthy retinal function, MG also have a prominent role during disease and after neuronal insult. In the mammalian retina, neuronal damage results in a MG gliotic response, involving the up‐regulation of stress proteins, proliferation and morphology changes (e.g. hypertrophy) (Bringmann & Wiedemann, 2012). This response is thought to be neuroprotective initially, but may ultimately culminate in dysfunction and death via loss of metabolic support or tissue integrity (Bringmann et al., 2009). While the mammalian retina lacks significant regenerative capacity (Jadhav et al., 2009; Ooto et al., 2004), it has been shown that re‐introduction of key molecular signals into MG can stimulate the genesis of new neurons in mice after acute damage (Jorstad et al., 2017, 2020; Karl et al., 2008), raising hopes that identifying the molecular mechanisms driving regeneration may provide therapeutic targets that we can exploit in the future. One such example is Hippo/Yap signalling, which has been identified as a signalling pathway that is altered after neuron degeneration and injury within MG cells (Hamon et al., 2017, 2019; Rueda et al., 2019).

Accordingly, intensive efforts have been made to uncover the molecular mechanisms regulating these endogenous regenerative responses in the retinas of “lower vertebrates”, such as fish and amphibians, where MG cells have the capacity to regenerate the retina after neuronal damage (Goldman, 2014). However, even though retinal regeneration is largely studied in these models, such as in the zebrafish (Fausett & Goldman, 2006; Fischer et al., 2002; Lenkowski & Raymond, 2014; Raymond et al., 2006; Thummel et al., 2008; Wan & Goldman, 2016), mechanisms are generally investigated in young adults, and regeneration is stimulated by inducing a high‐intensity acute damage injury, which is likely to differ from the slowly accumulating chronic damage associated with natural ageing. In the zebrafish retina, after high‐intensity acute damage, such as intense light or toxins (Lenkowski & Raymond, 2014; Thomas et al., 2012), MG undergo a brief reactive gliosis‐like phase that transitions into a regenerative response: a de‐differentiation and proliferation of the MG progenitor cell to specifically generate new neurons and restore vision (Eastlake et al., 2017; Thomas et al., 2016; Thummel et al., 2008). In contrast to high‐intensity acute injury, damage to the retina caused by degenerative disease in ageing will manifest itself over much longer timescales (wildtype zebrafish can live up to c.43 months in the lab (Carneiro et al., 2016)) and results in the gradual accumulation of DNA damage and cell death (Carneiro et al., 2016; Henriques et al., 2013; Salvi et al., 2006). As such, chronic degeneration in ageing may significantly differ from injury models, especially in its capacity to induce a regenerative response from glia. While multiple acute injuries do not appear to reduce the overall regenerative response of MG (Thomas et al., 2012), the effects of natural chronic age‐related damage on the MG regenerative potential has not been determined.

The zebrafish offers a suitable model for studying regeneration in the context of ageing, as it displays key human‐like hallmarks of ageing such as shorter telomeres and associated DNA damage (Carneiro et al., 2016). Accordingly, the well‐established *tert* ^−/−^ mutant zebrafish have impaired regeneration, both in steady‐state in tissues such as the gut (Anchelin et al., 2013; Henriques et al., 2013) and in response to injury, in the heart (Bednarek et al., 2015). The *tert* ^−/−^ model therefore offers the possibility of identifying telomerase‐dependent and telomerase‐independent molecular drivers of regeneration in a context of accelerated ageing. Moreover, the use of premature ageing models have provided key molecular insights into the mechanisms driving the ageing process and can contribute to the identification of therapeutic targets for age‐associated dysfunction and disease (Kubben & Misteli, 2017).

In this study, we used the naturally aged wildtype (WT) zebrafish and the prematurely aged *tert* ^−/−^ as models to study the impact of ageing and telomerase function on the maintenance of neuronal structure and regenerative capacity of MG cells. We started by confirming that naturally aged retinas display known hallmarks of retinal ageing, including tissue thinning, accumulation of DNA damage and neuronal loss (Van Houcke et al., 2019), which we show are accompanied by significant histopathological abnormalities in the retina. However, we now show that most of this damage is not accelerated in the absence of telomerase (*tert* ^−/−^ mutant), suggesting these to be largely telomerase‐independent. Interestingly, the accumulation of chronic damage with ageing is not sufficient to stimulate MG to proliferate or replace lost neurons. Instead, ageing leads to morphological and molecular aberrations in MG and loss of vision, recapitulating hallmarks of human retinal degeneration with advancing age. Moreover, we show that MG cells express Yap throughout lifespan, a key molecule involved in the regenerative response (Hamon et al., 2019; Rueda et al., 2019), but that this expression decreases with advancing age. Despite reduced Yap levels, aged MG cells are still capable of regenerating neurons in old zebrafish, after acute light injury. We, therefore, identify differences in the MG response to high‐intensity acute damage versus chronic damage, an important consideration for potential therapies aiming to stimulate endogenous regenerative mechanisms to treat human retinal disease.

## 2 RESULTS

### 2.1 The aged zebrafish retina displays neurodegeneration independently of telomerase

**FIGURE 1:**
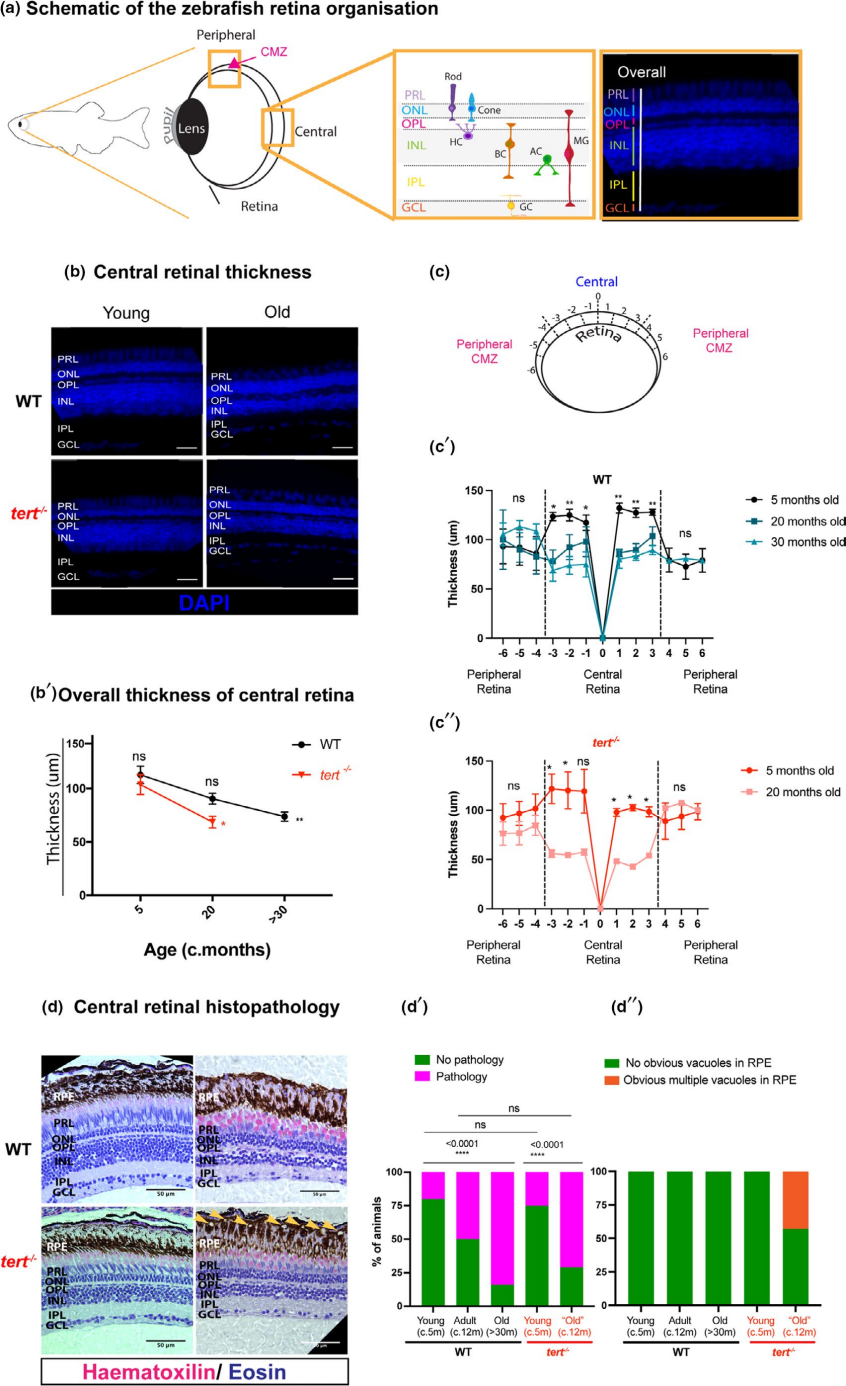
Zebrafish retina ageing is characterised by tissue thinning and morphological alterations, independently of telomerase. (a) Schematic figure of the zebrafish retina highlighting the peripheral, including the proliferative ciliary marginal zone (CMZ), and central retina, with respective layers and cell types (inset). (b) Central retina stained with DAPI in both WT and *tert* ^−/−^, at young (5 months) and old ages (>30 months in WT and c. 20 months in *tert* ^−/−^). Scale bars: 20 μm. (b’) Quantifications of retina thickness by DAPI staining. *N* = 3 per group. Error bars represent the standard error of the mean (SEM). (c) Schematic figure of the retina showing the twelve regions where retina thickness was quantified (6 central and 6 peripheral regions). (c’) Spider plots showing the thickness across the WT retina at 5 months, 20 months and 30 months. (c’’) Spider plots showing the thickness across the *tert* ^−/−^ retina at 5 and 20 months. (d) H&E staining in both WT and *tert* ^−/−^, at young (c. 5 months) and old ages (>30 months in WT and c. 12 months in *tert* ^−/−^). Scale bars: 50 μm. (d’–d’’) Qualitative assessment of H&E staining in the retina, (d’) considering overall pathology signs and (d’’) the presence of vacuoles in the RPE layer. *N* = 4–7 per group. GCL, ganglion cell layer; IPL, inner plexiform layer; INL, inner nuclear layer; OPL, outer plexiform layer; ONL, outer nuclear layer; PRL, photoreceptor layer. *p*‐value: *<0.05; **<0.01; ***<0.001

Similarly to humans, the zebrafish retina consists of three nuclear layers separated by two synaptic plexiform layers (Easter & Nicola, 1996). The nuclear layers consist in the outer nuclear layer (ONL), containing photoreceptors; the inner nuclear layer (INL) containing bipolar cells (BCs), amacrine cells (ACs), horizontal cells (HCs) and MG; and the ganglion cell layer (GCL) mainly containing retinal ganglion cells (RGCs) (see diagram in Figure 1a). Further, RGCs, ACs and BCs are neurons that come together to make connections in the major synaptic neuropil, called the inner plexiform layer (IPL; Figure 1a). The zebrafish central retina is considered fully specified by 73 h post‐fertilisation (hpf) (Easter & Nicola, 1996) and continually grows at the periphery throughout life due to proliferation of the ciliary marginal zone (CMZ) (Easter & Nicola, 1996). Thus, the central region of the adult retina can be considered the “oldest”, as it is largely generated during early developmental stages, and where there is very little proliferation to generate new neurons (Fu et al., 2018; Julian et al., 1998), with the exception of rods (Johns & Fernald, 1981; Julian et al., 1998; Nelson et al., 2008; Otteson et al., 2001). In contrast, the peripheral region of the retina is continually expanding at the margins due with the newest born cells being found adjacent to the CMZ (Van Houcke et al., 2019).

It has recently been shown that the ageing WT zebrafish retina shows degeneration phenotypes with advancing age (Fu et al., 2018; Van Houcke et al., 2019). Telomerase is known to be important for proliferation and regeneration in the zebrafish (Anchelin et al., 2013; Bednarek et al., 2015; Carneiro et al., 2016; Henriques et al., 2013); however, apart from its described role in the human retinal pigment epithelium (RPE) (Drigeard Desgarnier et al., 2016), its specific role in the ageing retina remains unclear. Here, we characterised key known hallmarks of human retina ageing (Eriksson & Alm, 2009), including retina thinning, morphology and histopathological alterations. We show that the WT and *tert* ^−/−^ zebrafish central retina progressively thins with ageing (Figure 1b,b’,c,c’,c’’), consistent with previous studies (Eastlake et al., 2017; Thomas et al., 2016). This retinal thinning is specific to the central retina, as the peripheral retina maintains its thickness over‐time (Figure 1c’,c’’ and Figure S1). Thinning of the central retina is accompanied by several morphological and histopathological alterations, including cellular spatial rearrangement, cellular atrophy and the development of vacuoles in the RPE (Figure 1c–c’’ and Figure S2). However, apart from specific alterations in the RPE, we show that this occurs largely independently of telomerase, as the telomerase‐deficient model *tert* ^−/−^ displays retinal thinning and most morphological and histopathological alterations at the same rate as the WT (Figure 1b’,c’).

**FIGURE 2:**
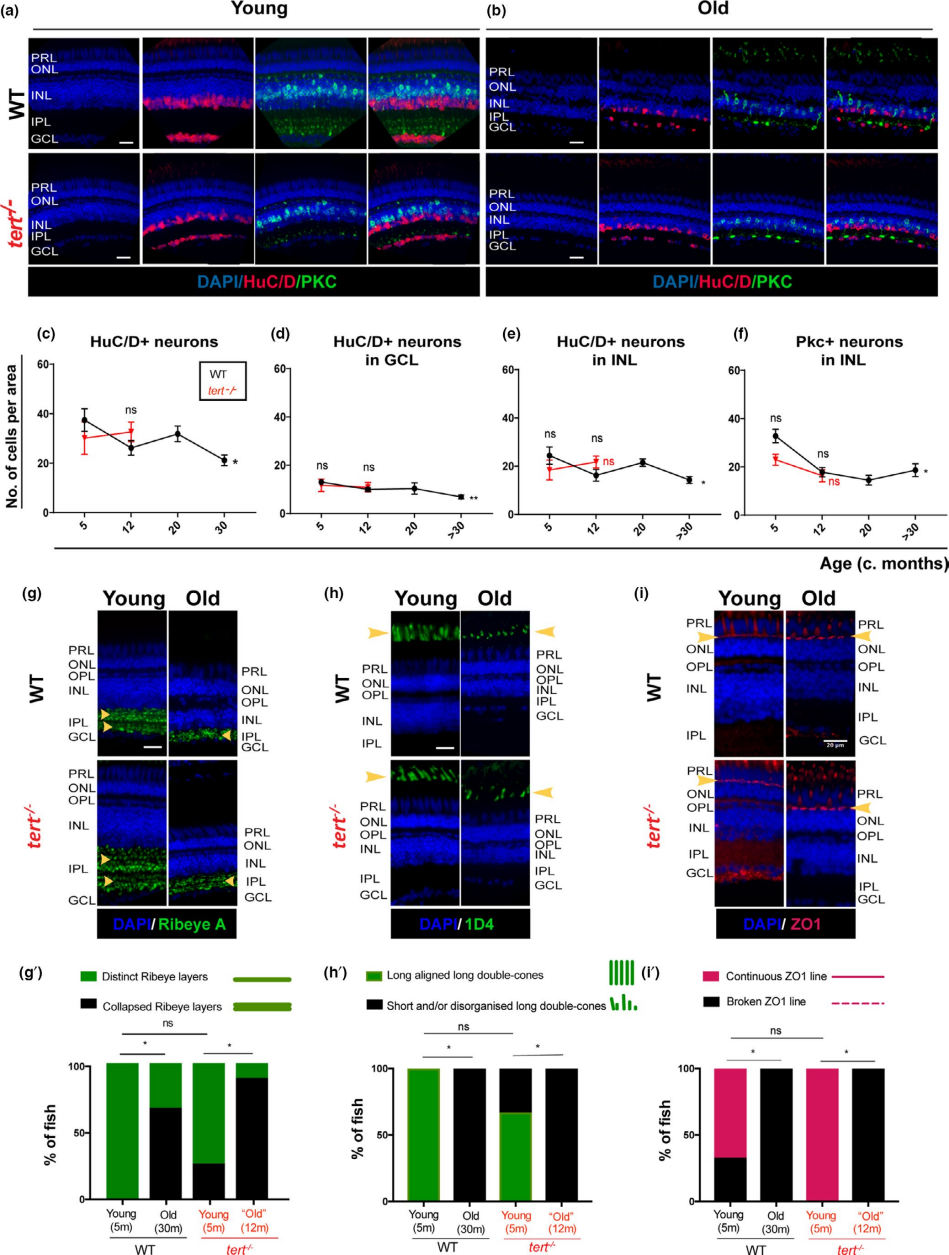
Zebrafish retina undergoes neurodegeneration with ageing, independently of telomerase. The central retina immunolabelled with HuC/D and PKC (amacrine in magenta and bipolar cells in green, respectively), in both WT and *tert* ^−/−^, in (a) young (5 months) and (b) old adults (>30 months in WT and 12 months in *tert* ^−/−^). Scale bars: 20 μm. (c–f) Quantifications of the number of HuC/D‐positive neurons per area (10,000 μm^2^) (c) in the overall retina, and (d) in the GCL (ganglion cells) and (e) INL (amacrine cells). (f) Quantifications of the number of PKC‐positive neurons cells per area (10,000 μm^2^) in the INL (bipolar cells). Error bars represent the SEM. *N* = 3–7. (g–i) The central retina immunolabelled with (g) Ribeye A (BC synaptic terminals, in green), (h) 1D4 (outer segments of long double‐cones, in green), and (i) ZO1 (outer limiting membrane, in red), in both WT and *tert* ^−/−^, in young (5 months) and old adults (>30 months in WT and 12 months in *tert* ^−/−^). Scale bars: 20 μm. (g’–i’) Quantification of the percentage of fish presenting defects in (g’) Ribeye A (phenotype observed as disorganised synaptic terminal layering in the IPL), (h’) 1D4 (short and/or misaligned long double‐cone outer segments), and (i’) ZO1 (broken outer limiting membrane). Error bars represent the SEM. (g’) *N* = 4–9, (h’, i’) *N* = 3–6

To determine which type of neuronal loss was underpinning retinal thinning, we used immunohistochemistry with several molecular markers specific for different neuronal populations from young to old ages, in the presence and absence of telomerase (*tert* ^−/−^ *)*. As expected, there is an overall reduction in the number of cells expressing several markers for retinal neurons, which indicates a decrease in neuron numbers with ageing in the zebrafish retina (Figure 2a–c). Specifically, we see decreased numbers of RGCs in the GCL (Figure 2d), HuC/D‐expressing ACs (Figure 2e), and PKC‐expressing BCs in the INL layer (Figure 2f). Additionally, in the aged zebrafish retina, BCs display disorganised axon terminals in the IPL (Figure 2a,b). Consistent with the retinal thinning, none of these neurodegenerative phenotypes are accelerated in the absence of telomerase. Thus, degeneration observed in these neuronal populations is expected to affect connectivity in the IPL. Previously, it has been shown that synaptic integrity is reduced in the ageing zebrafish retina (Van Houcke et al., 2019). Accordingly, we identified thinning of the synaptic IPL layer is accompanied by a reduction and disorganisation of Ribeye A‐positive BC terminals, suggesting a reduced number of synapses in the IPL with ageing (Figure 2g,g’). The loss of photoreceptor integrity is one of the key features of human retina ageing and disease (Lo, 2019) and we show that long double‐cones in the zebrafish retina undergo structural changes (short and/or disorganised) with ageing, shown by ID4 staining (Figure 2h,h’). To confirm the photoreceptor outer segment disorganisation, we stained rod and cone outer segments with the zpr3 antibody (Figure S3a) and red/green double cones with the zpr1 antibody (Figure S3b). Zpr3 staining clearly shows the shortened and misaligned outer segments in the WT aged retina, but is not accelerated in *tert* ^−/−^ (Figure S3a’), consistent with the ID4 staining. Furthermore, zpr1 staining shows not only a shortening of red/green cones in the WT aged retina (Figure S3b’), but also a reduced number of zpr1‐positive cells (Figure S3b’’), consistent with the previously reported reduction in cone and rod cell density with age (Van Houcke et al., 2019). These structural changes are also accompanied by disruption of the tight junction protein zonula occludens‐1 (ZO1) (Figure 2i,i’), a marker for the outer limiting membrane thought to be involved in photoreceptor degeneration (Fu et al., 2018). These defects are also not accelerated in the *tert* ^−/−^ at the ages tested, suggesting these are likely telomerase‐independent phenomena.

### 2.2 MG do not proliferate in response to chronic retinal neurodegeneration with ageing

**FIGURE 3:**
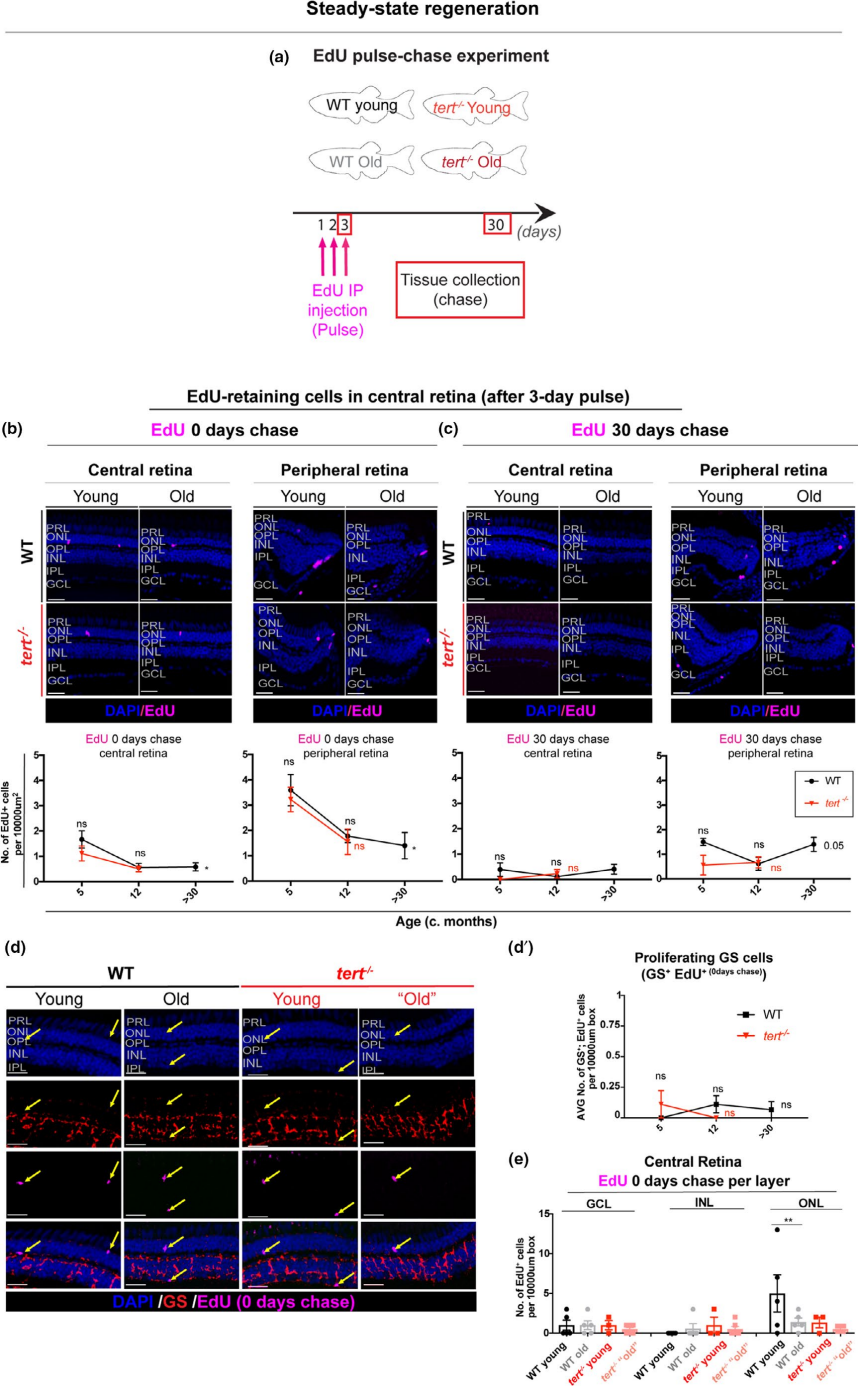
Aged zebrafish retina does not show signs of regeneration in response to spontaneous cell death and neuronal loss. (a) Schematic figure of the experimental design: 3‐day pulse of EdU, by IP injection, followed by 0‐ or 30‐day chase. We anticipate that in a healthy young fish the retina has some cells proliferating in the CMZ, which over‐time will replace older cells in the central retina. In the case of injury, we anticipate that there will be elevated levels of proliferation in peripheral and central retina to replace the dead cells. (b–c) The central and the peripheral retina immunolabelled with EdU (in purple), at (b) 0‐ and (c) 30‐day chase, in both WT and *tert* ^−/−^, in young (5 months) and old adults (>30 months in WT and 12 months in *tert* ^−/−^). Scale bars: 20 μm. Graphs show quantifications of the number of EdU‐retaining cells per area (10,000 μm^2^), in the overall central and peripheral retina at (b) 0‐ and (c) 30‐day chase. (d) The central retina immunolabelled with GS (Müller glia, in red) after a 3‐day pulse of EdU, by IP injection, at 0‐day chase, in both WT and *tert* ^−/−^, in young (5 months) and old adults (>30 months in WT and 12 months in *tert* ^−/−^). Scale bars: 20 μm. Yellow arrows show EdU‐positive cells. (d’) Quantifications of the number of GS‐positive; EdU‐positive cells per area (10,000 μm^2^). Error bars represent SEM. *N* = 3–6. (e) Quantifications of the number of EdU‐retaining cells per area (10,000 μm^2^), per layer of the retina, at 0‐day chase. Error bars represent SEM. *N* = 3–6

It is well‐established that MG in the zebrafish retina proliferate and regenerate lost neurons after high‐intensity acute damage (Jadhav et al., 2009; Jorstad et al., 2017; Ooto et al., 2004). Although the progressive age‐related retinal neurodegeneration would suggest a lack of regenerative response by MG, it remained unclear whether this degeneration was being counteracted, at least to some degree, by proliferation in the aged retina. To test this, we used a 5‐ethynyl‐2′‐deoxyuridine (EdU) pulse‐chase strategy to identify any cell divisions in the retina, from young to old ages, which would be indicative of potential regeneration. To characterise the steady‐state regenerative capacity of the central and peripheral CMZ until old age, we carried out a 3‐day pulse of EdU followed by 0‐ and 30‐day chase, in young and old WT and *tert* ^−/−^ zebrafish (Figure 3a). We observe few EdU‐positive cells at 0 day post‐chase and instead of a compensatory proliferation response, we detect even less EdU‐positive cells with ageing, suggesting overall reduced proliferative capacity in the central retina (Figure 3b). This was further confirmed by a reduced number of proliferating cell nuclear antigen (PCNA)‐positive cells in the natural aged zebrafish retina (Figure S4). These levels are further reduced after a 30‐day chase (Figure 3c), suggesting that there are very few cells proliferating in the aged central retina. In the peripheral retina, where the proliferative CMZ resides, we see double the EdU‐retaining cells in comparison to the central retina at 0‐day chase (Figure 3b). Nevertheless, proliferation in the peripheral retina (Figure 3b,c) is not increased with ageing, suggesting that there is no compensatory proliferation from the CMZ in response to neuronal loss with ageing. Finally, the fact that there are very low and decreasing levels of proliferation in wildtype ageing could explain why removing telomerase (*tert* ^−/−^) has no further significant effect. As proliferation of MG is the primary source of neurons after acute injury in fish (Bernardos et al., 2007; Fausett & Goldman, 2006), we wanted to determine whether there could be any small compensatory proliferation over‐time, within MG cells specifically. To do so, we co‐labelled cells with the MG specific marker glutamine synthetase (GS) and counted the GS‐positive; EdU‐positive cells. We detect very few GS‐positive; EdU‐positive cells in the central retina in both WT and *tert* ^−/−^, at any of the time‐points characterised throughout their lifespan (Figure 3d,d’), suggesting MG are not proliferating in the central retina at old ages. Thus, in contrast to what has been reported to occur in response to high‐intensity acute damage (Jadhav et al., 2009; Jorstad et al., 2017; Ooto et al., 2004), our data show that age‐related chronic cell death does not trigger proliferation of MG cells. Finally, rods can originate from rod‐specific progenitors into adulthood (Johns & Fernald, 1981; Julian et al., 1998), which are found in the ONL and are derived from MG that slowly divide in the WT retina (Bernardos et al., 2007; Nelson et al., 2008; Otteson et al., 2001; Raymond et al., 2006). To test whether there is any compensatory proliferation with ageing occurring specifically from rod‐specific progenitors found scattered throughout the ONL of the retina (Johns & Fernald, 1981; Stenkamp, 2015), we quantified levels of EdU‐positive cells in the different layers of the retina. Although we observe EdU‐positive cells in the central ONL of 5 months WT (likely to be rod precursors dividing), there are few detected in >30 months old WT. Once again, removing telomerase (*tert* ^−/−^ *)* has no further effect (Figure 3e). Together, our data show that there is no compensatory proliferation in response to age‐related degeneration in the zebrafish retina, by any of the known sources of regeneration and neurogenesis: CMZ, rod precursor cells or MG.

### 2.3 Zebrafish vision declines with ageing, independently of telomerase

**FIGURE 4:**
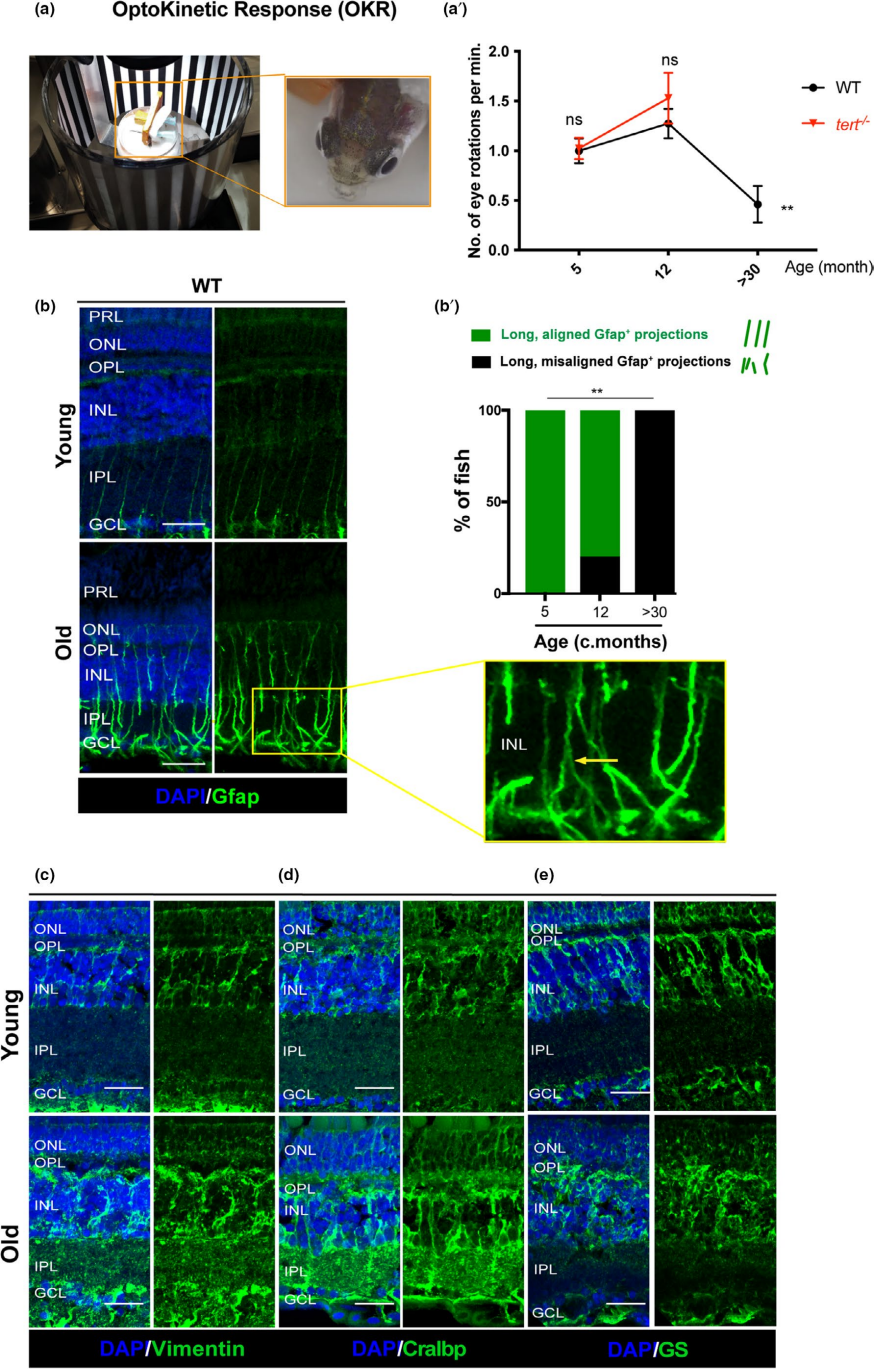
Zebrafish vision declines with ageing, which is accompanied by signs of glial morphological alterations. (a) OKR assay was performed by immobilising the fish in between soft sponges, inside a petri dish containing water, placed in the centre of a rotation chamber. The walls of the rotation chamber had 0.8 mm‐thick black and white stripes and the chamber was maintained at a constant velocity of 12 rpm throughout the experiment. (a’) The number of eye rotations per minute was manually quantified by video observation. Error bars represent the SEM. *N* = 5–8. (b) MG display signs of altered morphology at old ages, as evidenced by Gfap staining. The central retina immunolabelled with Gfap (in green), in WT, young (5 months) and old adults (>30 months). Yellow arrows highlight an example of aligned *versus* misaligned MG projections. (b’) Quantifications of the percentage of fish presenting disorganised MG processes in the IPL (detailed view‐inset). *N* = 6. (c–e) MG in the old retina show altered morphology and altered expression of stress proteins. The central retina immunolabelled with (c) Vimentin, (d) Cralbp, and (e) GS showing disorganised MG in old adults (>30 months) compared with young adults (5 months). *N* = 6. Scale bars: 20 μm

Loss of visual acuity and contrast sensitivity with advancing age in humans has been well documented (Marshall, 1987; Salvi et al., 2006). Here, we report that the presence of retinal neuron loss and tissue thinning in the aged retina is largely independently of telomerase (*tert*). Therefore, to determine whether the molecular and structural changes we observe have a pathological consequence on the retinal function, and whether this is accelerated in the absence of telomerase, we tested visual acuity in WT and *tert* ^−/−^ zebrafish, from young to old ages. Visual testing in the zebrafish has been used to screen for mutants with defects in retinal development and function (Brockerhoff et al., 1995; Gross et al., 2005; Lessieur et al., 2019; Neuhauss et al., 1999), but were yet to be tested in the context of ageing. The optokinetic response (OKR) is used to measure innate visual responses and provides readout of visual acuity (Brockerhoff et al., 1995; Tappeiner et al., 2012) (Figure 4a). Our results show that there is a decreased number of eye saccades *per* minute (min) in naturally aged fish (Figure 4a’ and Videos S1 and S2), suggesting that zebrafish vision indeed declines with ageing. Finally, telomerase does not seem to be a limiting factor for visual acuity, since the *tert* ^−/−^ zebrafish visual acuity decreases at the same rate as the WT (Figure 4a’ and Videos S3 and S4). As such, it does not appear that the telomerase‐dependent histopathological alterations in the retina significantly affect vision, and indeed loss of telomerase does not accelerate any of the hallmarks of zebrafish retina ageing tested here, unlike what is described in other tissues (Carneiro et al., 2016). We, therefore, focused on characterising the morphological and molecular changes underpinning the lack of MG regenerative responses in the ageing WT retina from here onwards.

### 2.4 MG become morphologically altered with ageing

MG respond to damage or injury in most, if not all, retinal degenerative diseases (Bringmann et al., 2009). In the mammalian retina alterations in MG morphology is a hallmark of MG response after retinal damage (Bringmann & Wiedemann, 2012), but also commonly observed in the aged retina (Telegina et al., 2018). The characteristic mammalian response is gliosis, whereby MG morphology becomes aberrant, accompanied by changes in expression and localisation of structural proteins like glial fibrillary acidic protein (Gfap) (Bringmann et al., 2009; Reichenbach & Bringmann, 2013). As we do not observe a MG regenerative response to neurodegeneration, but we do observe vision loss in naturally aged zebrafish, we wondered whether zebrafish MG were undergoing an ageing‐associated damage response similar to mammals. Here, we labelled MG using the well‐described GS antibody and characterised MG morphology and organisation using the markers Gfap, Vimentin and cellular retinaldehyde‐binding protein (Cralbp) (Garcia & Vecino, 2003; Verardo et al., 2008). Although the number of MG cells is maintained in the central retina throughout the zebrafish lifespan (Figure S5a), there is an obvious change in the stereotypical radial morphology of the MG cells in the WT aged retina (Figure 4b–e). Aberrations in the aged MG cells also include disruptions in their protrusions along the synaptic IPL and basal lamina (Figure 4b), which are known hallmarks of gliosis in retina degeneration (Telegina et al., 2018). Qualitative assessment of Gfap staining further shows that while all young fish display long and aligned MG basal processes, 100% of the old fish show morphological disorganisation of processes (Figure 4b’). We also observed aberrant MG shape and Vimentin, Cralbp and GS staining at old ages (Figure 4b–d). Thus, similarly to humans, chronic neurodegeneration with ageing correlates with altered MG morphology in the zebrafish retina rather than regeneration.

Microglia, the innate immune cells found in the retina (reviewed in Cragnolini et al., (2020)), are also key players in maintaining tissue homeostasis throughout life and part of the MG regeneration process (Conedera et al., 2019). They are activated in many neurodegenerative diseases, with increased numbers at sites of damage, including in the photoreceptor layer in many forms of retinal degeneration (reviewed in Telegina et al., (2018)). As we do not observe regeneration in the aged zebrafish retina in response to neuronal death, we asked whether there could be alterations in the number of microglia (4C4‐positive cells) found in the tissue. However, in contrast to what is observed in high‐intensity acute damage paradigms (Jorstad et al., 2017; Wan & Goldman, 2016), we observe few microglia in the central retina, with no increase with ageing. Furthermore, microglia did not appear to be localised to regions of neuronal death, such as in photoreceptors or the INL (Figure S5b,b’,b’’).

### 2.5 Müller glia express reduced levels of Yap in old age

**FIGURE 5:**
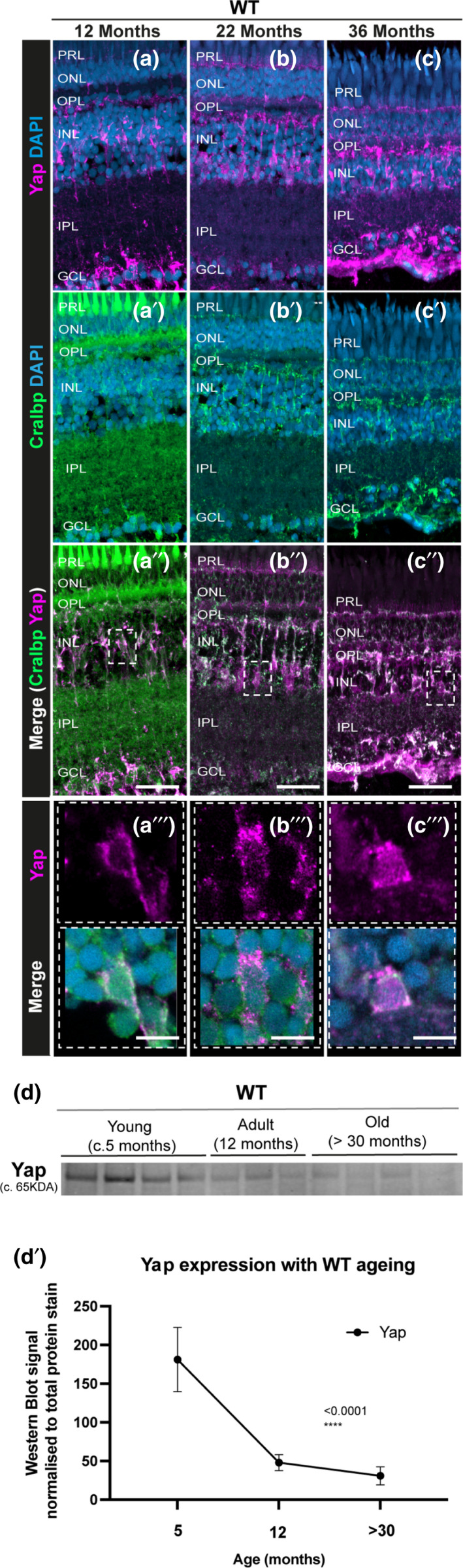
Yap expression is observed in the retinal MG throughout the zebrafish lifespan, but overall levels decrease with ageing. (a–c) The central retina immunolabelled for Yap expression (in magenta) and (a’–c’) Cralbp (MG cells, in green) in WT zebrafish at different ages (c. 12 months, 22 months, and 36 months). (a’’–c’’) Merge of DAPI, Yap and Cralbp staining and (a’’’–c’’’) respective inset showing Yap expression in MG cells at all stages observed. Representative images shown from *N* = 3 animals per age. Scale bars: 20 μm and 3 μm (in insets). (d–d’) Western blot of whole eyes and respective quantification show decreased Yap expression with ageing in WT zebrafish. *N* = 3–4

As we observe morphological alterations in MG cells and a lack of MG proliferation in response to age‐associated chronic damage, we next asked whether aged MG have altered molecular expression of Yap, a key molecule in the retinal regenerative process (Hamon et al., 2019). Specifically, when Hippo signalling is activated, LATS1/2 kinases phosphorylate the transcriptional co‐factor Yap, leading to its degradation. Importantly, Yap expression is required for MG regeneration (Hamon et al., 2019) and its expression goes up in reactive MG cells after acute damage (Rueda et al., 2019). We therefore tested whether Yap expression was affected in ageing and in MG specifically. Here we show, by immunohistochemistry, that Yap is expressed in MG until old age (Figure 5a–c), where it is thought to be required for the maintenance of MG proliferative capacity (Rueda et al., 2019). Nevertheless, overall expression levels in the eye are reduced, as assessed by western blot (Figure 5d,d’, and Figure S6). As such, MG retain the expression of a critical molecular player required for the regenerative process after damage, but its levels are significantly reduced. Given this, it was still unclear whether the naturally aged fish could regenerate their retina in response to a high‐intensity acute injury.

### 2.6 Müller glia retain the ability to regenerate after acute damage until old age

**FIGURE 6:**
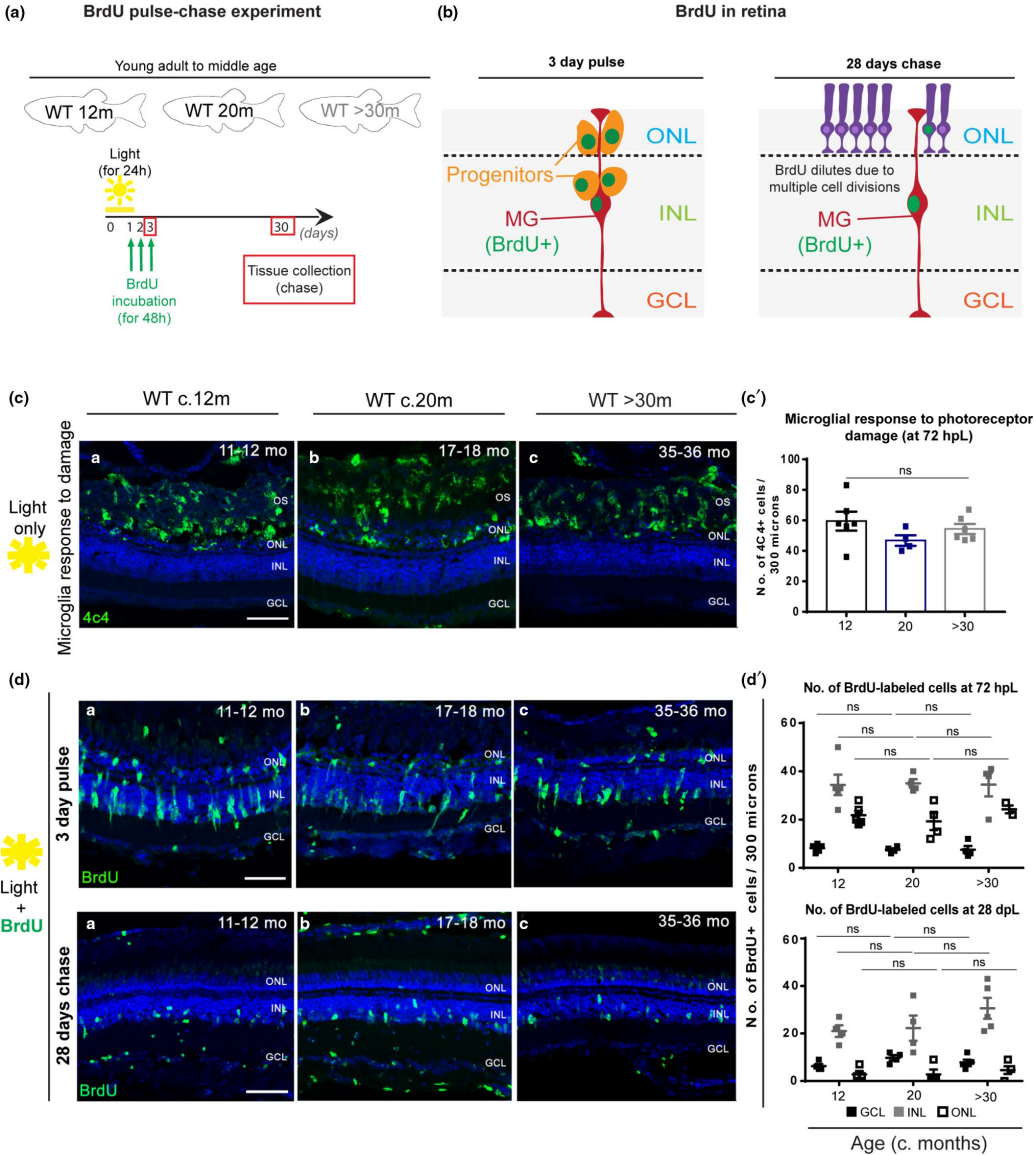
Zebrafish MG regenerative capacity upon acute damage is maintained in old age. (a) Schematic image of the experimental design and (b) expected results. Fish were light‐treated for 24 h and BrdU incorporation occurred between 24–72 h (green), which allowed for BrdU to be washed out the proliferating progenitors, leaving only MG which re‐entered the cell cycle to be labelled. (c) The central retina labelled with 4C4 (microglia, in green), after light treatment, in adults (c. 12 months), middle aged (c. 20 months) and old (>30 months) *albino* zebrafish. The majority of the microglia response to damage occurs within the OS and ONL of the retina, where a debris field is present due to photoreceptor degeneration. (c’) Quantification of the number of microglial cells which responded to photoreceptor damage in the different aged groups. (d, top) The central retina 72 h after light treatment onset, immunolabelled with BrdU (proliferation, in green). (d’). Quantification of the number of cells proliferating in the GCL (represented in black squares), INL (represented in grey squares), and ONL (represented in white squares) in each age group. (d, bottom) The central retina 28 days after light treatment onset. (d’). Quantification of the number of BrdU‐positive cells observed in the GCL, INL, and ONL of each aged group. Error bars represent the SEM. *N* = 4–5. Scale bars: 20 μm

Studies on retinal regeneration typically focus on the young adult retina and have not taken old age into account for the regenerative response. There is evidence from *ex vivo* mouse explants that MG neurogenic competence in response to neuronal death is limited with advancing age (Loffler et al., 2015); however, this study was restricted to early post‐embryonic stages and not more mature adult stages. Several high‐intensity acute damage paradigms induce retinal neuron death and elicit a regenerative response in zebrafish (Bernardos et al., 2007; Cameron, 2000; Hanovice et al., 2019; Ranski et al., 2018). Repeated damage to the adult retina, specifically between 9–18 months of age, which is still considered young for a WT fish, results in MG continuously re‐entering the cell cycle to proliferate and regenerate lost neurons, although they do show signs of chronic activation after repeated insults (Ranski et al., 2018). However, it remains unclear whether MG retain their ability to proliferate and regenerate the retina throughout life, and whether altered MG, such as those often occurring in retinal disease or old age retain proliferative capacity to replace damaged neurons. Since we show that MG retain Yap expression into old ages, albeit at reduced levels, it remained possible that aged zebrafish retinas could still regenerate in response to high‐intensity acute damage. To test this, we used the light‐damage model where aged *albino* zebrafish, at three stages of their lifespan, were treated with light to elicit photoreceptor damage. To detect increased proliferation of MG in response to damage, a 3‐day pulse of bromodeoxyuridine (BrdU) was performed, followed by a 28‐day chase period (Figure 6a). After a 3‐day pulse, BrdU should label both MG and their daughter cells, the newly formed progenitors (Figure 6b). However, since the BrdU staining dilutes in the rapidly dividing progenitor cells and MG only divide once, after a 28‐day chase, the majority of the BrdU staining is retained in MG (Figure 6b). Our results show that light‐lesion in aged retinas leads to a loss of photoreceptors, which is accompanied by a strong increase in microglia in the ONL, typical of photoreceptor degeneration in this damage model (Figure 6c,c’). Moreover, there are no differences in the incorporation of BrdU with ageing on any layer, at 72 h post‐light damage (hpL), when the initial regenerative response is mounted, or at 28 days post‐light damage (dpL), when the number of MG that initially re‐entered the cycle can be identified (Figure 6d,d’). Both the unaltered immediate timing of MG response and the overall capacity to regenerate each neuronal layer are maintained with increased age. Finally, MG in the aged *albino* background also show a characteristic gliosis phenotype similar to WT (Figure S7). Together, these results suggest that the regenerative response remains intact throughout zebrafish lifespan and the molecular and morphological alterations in aged MG does not impact their regenerative capacity in old age.

## 3 DISCUSSION

Using a combination of cell labelling strategies throughout adulthood into old age, we show that the zebrafish retina retains its potential to regenerate in response to acute damage into old age. Therefore, the lack of compensatory proliferation in response to chronic, age‐associated cell death in the ageing zebrafish retina is not due to a loss of capacity of MG to proliferate *per se*, but likely due to the absence or insufficient levels of the required stimuli for MG proliferation and regeneration.

### 3.1 Telomerase‐dependent and telomerase‐independent hallmarks of zebrafish retinal ageing

Previous work has suggested that telomerase and telomere length are important for human retinal health. In particular, the RPE has been reported to have shorter telomeres than the neural retina, and it accumulates senescence over time (Drigeard Desgarnier et al., 2016). Accordingly, reactivation of telomerase has been described to ameliorate symptoms of age‐associated macular disease (Dow & Harley, 2016; Rowe‐Rendleman & Glickman, 2004) and telomerase activators are currently in clinical trials (e.g. NCT02530255). However, retinal homeostasis requires more than RPE maintenance. It requires a steady‐state level of proliferation of different cell types involved in multiple aspects of retina function. As in other proliferative tissues (Anchelin et al., 2013; Carneiro et al., 2016; Henriques et al., 2013), it would be expected that telomerase levels would influence zebrafish retina homeostasis. We, therefore, hypothesised that the zebrafish retina would degenerate in a telomerase‐dependent manner, leading to vision loss. However, our data suggest that, apart from aberrations in the RPE, most age‐related changes described here in the naturally aged zebrafish retina, including vision loss, are largely telomerase‐independent, since depletion of telomerase (*tert* ^−/−^) does not accelerate or exacerbate any of these phenotypes. Telomere dysfunction is known to affect mostly highly proliferative tissues (reviewed in Henriques and Ferreira, (2012)) and what our data show is that, in the region most affected by ageing phenotypes, the central retina, there is very little proliferation to start with, potentially explaining why telomerase depletion has no further significant effect.

### 3.2 Chronic vs. high‐intensity acute damage in MG responses: a “tipping point” required to stimulate regeneration in ageing?

Regeneration studies so far have relied on several damage paradigms, including phototoxic (Ranski et al., 2018) and ouabain‐induced lesions (Mitchell et al., 2019), which induce rapid cell death post‐insult. While high‐intensity acute damage models are suitable to explore the cellular and molecular mechanisms underpinning the regenerative potential of the retina, they do not test whether this regenerative response is also occurring with natural ageing in the retina. Our results show that zebrafish develop vision loss with ageing and that this is accompanied by retinal neurodegeneration and MG morphological and molecular aberrations, rather than proliferation and replacement of dying neurons. This is counterintuitive to what would be expected based on the well‐known regenerative response to neuronal death by MG. Nevertheless, we show that aged zebrafish retinas maintain their regenerative response and capacity after acute injury. This study, therefore, shows that there are critical differences between the MG regenerative response to the neuronal loss induced by chronic damage in ageing and after high‐intensity acute injury.

“Natural ageing” and associated neuronal loss play out over months or even years and we now show that this does not elicit the same regenerative response as high‐intensity acute damage. This may be because the slow degeneration is not producing a strong enough signal to induce MG to undergo the cellular and molecular processes of regeneration. It has been shown that the level of cell death can induce a differential response of MG cells. Whilst a large amount of rod death causes a regenerative response, small amounts do not (Lessieur et al., 2019; Montgomery et al., 2010; Thomas et al., 2012). Furthermore, there may be distinct differences in signals released after apoptosis or necrosis in ageing *vs* damage models (Bergmann & Steller, 2010; Nelson et al., 2013). In support of this concept, recent work suggests that there may be key signalling differences underpinning the difference between a “regenerative” or a “reparative” response to injury (Conedera et al., 2020). Thus, we propose that a molecular signal, or expression changes of such signal, will regulate the “tipping point” required to elicit a MG regenerative response in ageing.

Research in the context of acute damage paradigms in the zebrafish retina has uncovered many of the molecular mechanisms regulating this regenerative response (reviewed in Goldman, 2014; Julian et al., 1998). For instance, proliferation appears to be a key mechanism for the initiation of the regenerative response as blocking proliferation after damage in the zebrafish retina results in MG alterations, and not regeneration (Thummel et al., 2008), similarly to humans. Moreover, age‐associated degeneration of retinal neurons and their synapses, as we observed to occur in this study, may result in a loss of neurotransmitter release, such as gamma‐aminobutyric acid (GABA), which has been shown to facilitate the initiation of MG proliferation (Rao et al., 2017). Thus, dysregulation of neurotransmitters upon neurodegeneration could inhibit the key molecular pathways regulating regeneration. Alternatively, the initial inflammatory response has also been shown to be determinant for the repair process. Upon light‐induced retinal damage, overexpression and subsequent release of tumour necrosis factor (TNF) by apoptotic photoreceptors seems to induce MG proliferation (Iribarne et al., 2019; Thomas et al., 2016). After high‐intensity acute damage, there is also an increased number of microglia in the retina (Mitchell et al., 2018, 2019) and they have been shown to be essential for retinal regeneration (Conedera et al., 2019; White et al., 2017). In contrast, we show that microglia numbers do not increase with ageing in the zebrafish retina, and therefore, may compromise the regenerative response. Nonetheless, it is important to consider that the available immunohistochemistry techniques to identify microglia numbers have a few limitations. Namely, there may be a spike in microglia number in the aged retina that is rapidly resolved and missed at our time points or microglia may undergo apoptosis similar to retinal neurons. Thus, we cannot exclude the possibility that microglial cells are involved in the observed retinal degenerations or play a critical role in the lack of a regenerative response observed in the aged retina. However, identifying this regeneration signal, and the level required for inducing the response, will be critical to stimulate regeneration in the human retina. It is very promising that mammalian MG can be molecularly manipulated to increase their regenerative potential after damage (Jorstad et al., 2017). However, for these therapies to be truly transformative for human blinding conditions this regenerative capacity must be paired with the appropriate “damage” stimuli since using acute light damage or toxins in humans is unlikely to be therapeutically feasible.

### 3.3 MG response to chronic damage in ageing

The regenerative capacity of vertebrate tissues tends to decrease after repeated injury and as animals advance in age (Ranski et al., 2018). In the context of the central nervous system, this is aggravated by gliosis, a reactive change in glial cells in response to damage. In the mammalian retina, MG cells undergo gliosis in response to damage and in many retinal degenerative diseases (Bringmann et al., 2009). However, in the zebrafish retina MG cells undergo the initial reactive gliotic response after acute damage but quickly shift to the regenerative pathway. Here, we show that while we observe morphological and molecular alterations in MG cells in response to age‐related neurodegeneration, MG in the aged retina retain their ability to regenerate in response to high‐intensity acute damage of photoreceptors. This provides evidence that regenerative mechanisms in tissues that have been damaged or are degenerating remains possible. Importantly, the fact that MG are still capable of proliferating in response to a high‐intensity acute injury in old animals, shows that the lack of regeneration in the context of ageing is not due to an intrinsic inability of MG to proliferate *per se*, suggesting that it is the stimuli itself that is different/insufficient in the context of ageing. Mechanistically, in contrast to what has been described in high‐intensity acute damage models, where Yap levels go up (Rueda et al., 2019), we show that overall Yap levels decrease in the chronic damage setting of ageing. It is, therefore, a likely possibility that at least one of the key stimuli missing may be one that is responsible for switching off Hippo signalling pathway, preventing Yap degradation.

## 4 CONCLUSIONS

Our work demonstrates that, in the context of age‐induced neuronal degeneration, zebrafish MG undergo degeneration, rather than regeneration. This resembles what occurs in the aged human retina as well as in many human retinal degenerative diseases. Importantly, we identify key differences between chronic versus acute damage and show that aged, aberrant MG cells can be stimulated to repair damaged neurons in the old zebrafish retina.

## 5 MATERIALS AND METHODS

### 5.1 Zebrafish husbandry

Zebrafish were maintained at 27–28°C, in a 14:10 h (h) light–dark cycle and fed twice a day. The OKR was performed in the University College London (UCL) Institute of Ophthalmology and the phototoxic lesions and regeneration experiments were performed at Wayne State University School of Medicine (USA). All other experiments were performed in the University of Sheffield. All animal work was approved by local animal review boards, including the Local Ethical Review Committee at the University of Sheffield (performed according to the protocols of Project Licence 70/8681) and the Institutional Animal Care and Use Committee at Wayne State University School of Medicine (performed according to the protocols of IACUC‐19‐02‐0970).

### 5.2 Zebrafish strains, ages and sex

Three strains of adult zebrafish (*Danio rerio*) were used for these studies: wildtype (WT; AB strain), *tert* ^−/−^ (*tert^AB/hu3430^*) and *albino* (*slc45a2^b4/b4^*), each of mixed sexes. Wildtype (WT; AB strain) and albino (*slc45a2^b4/b4^*) have been obtained from the Zebrafish International Resource Center (ZIRC).

The *telomerase* mutant line *tert^AB/hu3430^* was generated by *N*‐ethyl‐nitrosourea mutagenesis (Utrecht University, Netherlands; Wienholds, 2004). It has a *T*→*A* point mutation in the *tert* gene and is available at the ZFIN repository, ZFIN ID: ZDB‐GENO‐100412‐50, from ZIRC. The fish used in this study are direct descendants of the ones used previously (Carneiro et al., 2016; Henriques et al., 2013), by which point it had been subsequently outcrossed five times with WT AB for clearing of potential background mutations derived from the random ENU mutagenesis from which this line was originated. The *tert^hu3430/hu3430^* homozygous mutant is referred to in the paper as *tert* ^−/−^ and was obtained by incrossing our *tert^AB/hu3430^* strain. Genotyping was performed by PCR of the *tert* gene (Carneiro et al., 2016; Henriques et al., 2013). The telomerase null mutant (*tert* ^−/−^ *)* zebrafish, extensively characterised elsewhere (Anchelin et al., 2013; Carneiro et al., 2016; Henriques et al., 2013), displays no telomerase activity and has significantly shorter telomeres from birth, ageing and dying prematurely (Carneiro et al., 2016). While *tert* ^−/−^ fish have a lifespan of 12–20 months, WT fish typically die between 36–42 months of age (Carneiro et al., 2016; Henriques et al., 2013). In order to study age‐related phenotypes in the zebrafish retina, in this study, we use an age >30 months old fish for what we consider old in WT (in the last 25–30% of their lifespan), and we consider the *tert* ^−/−^ old fish at the equivalent age (>12 months) that corresponds to the last 25–30% of their lifespan, approximately. In specific, “Old” was defined as the age at which the majority of the fish present age‐associated phenotypes, such as cachexia, loss of body mass and curvature of the spine. These phenotypes develop close to the time of death and are observed at >30 months of age in WT and at >12 months in *tert* ^−/−^ (Carneiro et al., 2016; Henriques et al., 2013). In addition, we used adult *albino* zebrafish for retinal regeneration studies (described in detail below). Importantly, none of the animals included in this study displayed visible morphological alterations in the eyes (e.g. cataracts).

### 5.3 Optokinetic response (OKR) assay

Fish were anaesthetised in 4% tricaine methanesulfonate (MS‐222; Covetrus, pharmaceutical grade) and placed in a small bed‐like structure made of sponge, located inside a small petri dish containing fish water. During the experiment, fish were maintained still by the strategic use of needles that sustained the sponges close to the fish so that the fish could not move. The petri dish was then placed inside a rotation chamber with black and white striped walls (8 mm‐thick stripes). After fish recovered from anaesthesia, the trial began, and the walls of the chamber started rotating at 12 rotations *per* min (rpm; for 1 min to the left side followed by 1 min to the right side). Eye movements were recorded using a digital camera throughout the experiment. After the experiment, the number of eye rotations *per* minute was measured by video observation and manually counting. The counting was performed blindly by two independent researchers. In the end, the results were normalised for the WT young from the same day/batch, in order to control for different days of experiments.

### 5.4 Intense light‐damage paradigm with BrdU incorporation

A photolytic damage model in adult *albino* zebrafish was utilised to destroy rod and cone photoreceptors and elicit a regenerative response (Thomas et al., 2012). Briefly, adult *albino* zebrafish were dark‐adapted for 10 days prior to a 30 min exposure to ~100,000 lux from a broadband light source. Next, fish were exposed to ~10,000 lux of light from four, 250 W halogen lamps for 24 h. Following 24 h of light treatment, fish were transferred to a 1 L solution containing 0.66 g of NaCl, 0.1 g Neutral Regulator (SeaChem Laboratories, Inc.), and 1.5 g BrdU (5mM; B5002; Sigma‐Aldrich) for 48 h. This timeframe for BrdU incubation was based on previous studies in order to label MG cell‐cycle re‐entry (Garcia & Vecino, 2003). Following a 48‐h incubation in BrdU, the fish were split into two groups: one group was euthanised by an overdose of 2‐Phenoxyenthanol and eyes were processed for immunohistochemistry as described below; the second group returned to normal husbandry conditions for an additional 25 days (or 28 days after light onset) prior to euthanasia and tissue collection. During this time, BrdU incorporation dilutes in actively dividing cells, allowing for clear visualisation of the number of MG cells in the INL that only divide a single time. It also serves as an indirect measure of regenerative capacity (i.e. an equal loss of BrdU‐positive cells at 28 dpL between experimental groups would indicate similar numbers of progenitor cell divisions earlier in the regenerative process).

### 5.5 Tissue preparation: paraffin‐embedded sections and cryosections

Adult fish were culled by overdose of MS‐222, followed by confirmation of death. Whole fish or dissected eyeballs were then processed for paraffin‐embedded sections or for cryosections. Importantly, all quantitative and qualitative comparisons presented in figures were performed in like‐for‐like (i.e. paraffin *versus* paraffin and cryostat *versus* cryostat sections). Paraffin sections were used for Figures 1, 2, 3 and Figures [Link], [Link], [Link], [Link], [Link]. Cryosections were used for Figures 4, 5, 6 and Figure S7.

Paraffin‐embedded sections. Whole fish were fixed in in 4% paraformaldehyde (PFA) buffered at pH 7.0, at 4°C for 48–72 h, decalcified in 0.5 M ethylenediaminetetraacetic acid (EDTA) at pH 8.0 for 48–72 h, and embedded in paraffin by the following series of washes: formalin I (Merck & Co) for 10 min, formalin II for 50 min, ethanol 50% for 1 h, ethanol 70% for 1 h, ethanol 95% for 1 h 30 min, ethanol 100% for 2 h, ethanol 100% for 2 h 30 min, 50:50 of ethanol 100%: xilol for 1 h 30 min, xylene I for 3 h, xylene II for 3 h, paraffin I for 3h and paraffin II for 4 h 30 min. Paraffin‐embedded whole fish were then sliced in sagittal 4 μm‐thick or coronal 16 μm‐thick sections, using a Leica TP 1020 cryostat. These sections were then used for immunohistochemistry and haematoxylin and eosin staining.

Cryopreservation and cryosections. Dissected eyeballs were fixed in 4% PFA at 4°C, overnight (ON). Then, they were washed in cold 1× phosphate‐buffered saline (PBS) and immersed in 30% sucrose in PBS, ON at 4°C, for cryopreservation. Single cryopreserved eyeballs were then embedded in mounting media—optimal cutting temperature compound (OCT, VWR International)— snap‐frozen in dry ice, and stored at −20°C until cryosectioning. Cryosections were sliced at a 13 μm thickness using a Leica Jung Frigocut cryostat or a Leica CM1860 cryostat. These sections were then used for immunohistochemistry.

### 5.6 Immunohistochemistry (IHC)

Before immunofluorescence staining, cryosections were hydrated in PBS at room temperature (RT) for 10 min, and paraffin‐embedded sections were deparaffinised and hydrated as follows: histoclear (Scientific Laboratory Supplies, Wilford) 2× for 5 min, followed by ethanol 100% 2× for 5 min, ethanol 90% for 5 min, ethanol 70% for 5 min and distilled water 2× for 5 min. After antigen retrieval in 0.01 M citrate buffer at pH 6.0 for 10 min, the sections were permeabilised in PBS 0.5% Triton X‐100 for 10 min and blocked in 3% bovine serum albumin (BSA), 5% Goat Serum (or Donkey Serum), 0.3% Tween‐20 in PBS, for 1 h. The slides were then incubated with the primary antibody at 4°C ON. After washes in PBS 0.1% Tween‐20 (3× 10 min) to remove excess to primary antibody, the sections were incubated with secondary antibody at RT for 1 h. Finally, the slides were incubated in 1 μg/ml of 4′,6‐diamidino‐2‐phenylindole (DAPI, Thermo Fisher Scientific) at RT for 10 min, washed in PBS 1×, and mounted with vectashield (Vector Laboratories). The primary and secondary antibodies used in this study are described in Tables 1 and 2 respectively.

**TABLE 1:** Primary antibodies used for immunostaining

Antibody, species and type	Dilution factor	Catalogue number; Company, City, Country
PCNA mouse monoclonal	1:500	NB500‐106; Novus Biologicals, Littleton, CO, USA
PCNA rabbit polyclonal	1:50	GTX124496; GeneTex, Irvine, CA, USA
7.4.C4 (4C4) mouse monoclonal	1:100	A gift from A. McGown
HuC/D mouse monoclonal	1:100	A21271; Thermo Fisher Scientific, Waltham, MA, USA
PKCβ1 rabbit polyclonal	1:100	Sc−209; Santa Cruz, Dallas, TX, USA
Ribeye A rabbit polyclonal	1:10,000	A gift from Teresa Nicholson
Gfap rabbit polyclonal	1:200	Z0334, Agilent DAKO, Santa Clara, CA, USA
Gfap mouse monoclonal	1:100	zrf1, ZIRC
Glutamine Synthase (GS) mouse monoclonal	1:150	mab302, Merck, Kenilworth, NJ, USA
1D4 rabbit polyclonal	1:5,000	A gift from David Hyde
BrdU rat	1:200	OBT0030A, Accurate Chemical & Scientific, Westbury, NY, USA
Yap mouse monoclonal	1:200	Sc‐101199; Santa Cruz, Dallas, TX, USA
Cralbp rabbit polyclonal	1:200	15356‐1‐AP; Proteintech, Rosemont, Illinois, USA
Vimentin mouse monoclonal	1:500	60330‐1‐IG; Proteintech, Rosemont, Illinois, USA
Zpr1 mouse monoclonal	1:50	ZIRC
Zpr3 mouse monoclonal	1:50	ZIRC
Zona Occludins 1 (ZO1)	1:100	ZO1‐1A12

**TABLE 2:** Secondary antibodies used for immunostaining

Antibody, species and type	Dilution factor	Catalogue number; Company, City, Country
Goat anti‐rabbit IgG Alexa Fluor^®^ 488	1:500	A11008; Invitrogen, Carlsbad, CA, USA
Goat anti‐rat IgG Alexa Fluor^®^ 488	1:500	A11006; Thermo Fisher Scientific, Waltham, MA, USA
Goat anti‐rabbit IgG Alexa Fluor^®^ 568	1:500	10032302; Thermo Fisher Scientific, Waltham, MA, USA
Donkey anti‐rabbit IgG Alexa Fluor^®^ 647	1:500	A31573; Thermo Fisher Scientific, Waltham, MA, USA
Goat anti‐mouse IgG Alexa Fluor^®^ 488	1:500	A11001; Thermo Fisher Scientific, Waltham, MA, USA
Goat anti‐mouse IgG Alexa Fluor^®^ 568	1:500	10348072; Thermo Fisher Scientific, Waltham, MA, USA
Goat anti‐mouse IgG Alexa Fluor^®^ 647	1:500	A21235; Thermo Fisher Scientific, Waltham, MA, USA
IRDye 800CW Goat anti‐Mouse IgG	1:10,000	926‐32210; Li‐COR, Nebraska

### 5.7 Haematoxylin & Eosin (H&E) staining

Haematoxylin & Eosin staining was performed in paraffin‐embedded sections as follows. Sections were de‐waxed and hydrated through a sequence of washes in xylene (2× 5 min), 100% ethanol (2× 5 min), 95% industrial methylated spirit (IMS; 5 min), 70% IMS (5 min), and water (1 min). Then, the tissues were incubated in Gill's II haematoxylin (VWR International, Pennsylvania) for 2 min and in 1% aqueous Eosin Y solution (Sigma‐Aldrich, Missouri) for 5 min. Finally, the sections were dehydrated (70% IMS for 10 s, 95% IMS for 10 s, 100% ethanol for 1 min, and xylene for 4 min) and mounted using dibutylphthalate polystyrene xylene (DPX) mounting medium (Merck, Kenilworth).

### 5.8 Ethynyl‐2′‐deoxyuridine (EdU) labelling

EdU labelling was detected using the Click‐iT^®^ EdU Imaging Kit (Thermo Fisher Scientific), following manufacturer's instructions. Briefly, fish were injected with 5 μl of 10 mM EdU diluted in dimethyl sulfoxide (DSMO), by intraperitoneal (IP) injection, for 3 consecutive days (3‐day pulse). In order to differentiate proliferating cells and low‐proliferative EdU‐retaining cells, the fish were separated into two groups: 0‐day chase and 30‐day chase groups. The fish from the first group were culled 2 h 30 min after the last injection of Edu, whereas the fish from the second group were culled 30 days after the last injection of EdU. After culling, whole fish were processed for paraffin‐embedded sections as described above. In order to detect EdU labelling in paraffin‐embedded sections, the slides were deparaffinised, hydrated, underwent antigen retrieval, were permeabilised and washed in 1× PBS. The slides were incubated in freshly made EdU‐labelling solution (*per* 1 ml of solution: 860 μl of 1× Click‐iT^®^EdU reaction buffer, 40 μl of CuSO_4_, 2.5 Alexa Fluor^®^ 647 azide working solution, and 100 μl of 10× EdU reaction buffer additive) at RT for 30 min. Finally, the slides were washed in 1× PBS before blocking and incubation with primary antibody (ON, at 4°C). The incubation in the secondary antibody was performed as previously described.

### 5.9 Imaging and quantifications

Paraffin‐embedded sections were imaged by epifluorescence microscopy, using a DeltaVision microscope with a 40× oil objective. Cryosections were imaged by laser scanning confocal imaging, using a Leica SP5 microscope, Nikon A1 Confocal microscope or a Zeiss 900 Airyscan 2 confocal, with a 40× oil objective. In either paraffin or cryosections, multiple 0.2–0.6 μm thick *z*‐stacks were acquired in order to capture the whole retina region. For each staining, a total of 4 images were taken per retina, 2 from central and 2 from peripheral retinal. The central retina was defined to be the centre point between opposing CMZs (a minimum of ~1000 μm from the periphery). The peripheral retina was defined as the tissue directly adjacent to the CMZ.

In order to quantify the alteration in the staining patterns, a z‐projection was generated using ImageJ (Rasband, W.S., ImageJ, U. S. National Institutes of Health, https://imagej.nih.gov/ij/, 1997–2018.) and three boxes of 100 × 100 μm were drawn in each field of view. Retinal thickness was measured in DAPI stained paraffin sections, using the software ImageJ to measure the individual layers and overall layers thickness as indicated in the diagram in Figure 1a. For key protein staining, the total number of positive cells was manually counted for each label.

Ribeye A, 1D4, ZO1, ZPR3 and Gfap staining were an exception to this, for which a qualitative assessment was performed instead. To do so, structural and morphological defects were identified as follows. For the outer segments of long double‐cones (1D4) staining, as young WT retinas usually display long and organised outer segments, all short and/or misaligned outer segments were considered defective. For the ZO1 staining, the average number of breaks in the ZO1‐labelled membrane *per* animal was quantified. The average of breaks in the WT young animals was used as a reference, and any fish presenting an average number of breaks below this average was considered to present defects in the membrane. For the Ribeye A staining, young retinas usually present two distinguished layers of pre‐synaptic ribbons. Thus, staining where the two layers of pre‐synaptic ribbons are not distinguished, was considered defective. Gfap staining usually reveals long and aligned MG processes in young WT retinas, and therefore, short and/or misaligned MG processes are considered altered. This qualitative assessment was used to calculate the percentage of fish *per* group presenting structural and morphological defects. An equivalent qualitative assessment was performed on Zpr3 staining. Zpr1 staining was used to identify red green cones, and these were counted manually and their length was measured using image J (Figure S3).

Finally, raw images were used for quantification purposes. The images were then processed with Adobe Illustrator 21.0.2 for display purposes.

### 5.10 Protein extraction, quantification and Western blotting

Adult fish were culled by overdose of MS‐222, followed by confirmation of death. Dissected eyeballs were snap‐frozen in liquid nitrogen and kept at −80°C until protein extraction, as follows. Frozen tissue was homogenised in 100 μl of radioimmunoprecipitation assay (RIPA) buffer (150 mM Sodium Chloride, 0.5% Sodium deoxycholate, 1% Triton‐X‐100, 0.1% SDS, 50 mM Tris‐HCL pH 8 (all from Sigma‐Aldrich) with Halt(TM) Protease and Phosphatase Inhibitor (Thermo Fisher Scientific)), by using a mechanical homogenizer (VWR International) and pestles (ThermoFisher Scientific) for approximately 30 s on ice. Samples were then incubated on ice for 30 min before spun down at 4°C, 13,000 rpm, for 10 min. The supernatant was collected, snap‐frozen in liquid nitrogen and kept at −80°C until needed.

Protein quantification was performed using the biochonic acid (BCA) assay kit (ThermoFisher Scientific), according to manufacturer's instructions. A total of 30 μg of protein were loaded *per* test sample were prepared with Bolt™ 1× lithium dodecyl sulphate (LDS) Sample Buffer and Bolt™ Sample Reducing Agent (Thermo FisherScientific) and denaturing at 70°C for 10 min before loading on to Invitrogen™ Bolt™ 4–12% Bis‐Tris Plus Gels, 12 wells, using the mini‐gel tanks (Thermo Fisher Scientific) according to manufacturer's instructions. Briefly, samples were run at 200 V for 25–40 min, in 1X compound 2‐(N‐morpholino) ethanesulfonic acid (MES) sodium dodecyl sulphate (SDS) running buffer with added Bolt™ Antioxidant (Thermo Fisher Scientific) alongside the Chameleon^®^ Duo Pre‐stained Protein Ladder (Li‐COR), and transferred using Invitrogen™ Novex™ iBlot™ polyvinylidene difluoride (PVDF) Transfer Stacks (Invitrogen), and the iBlot 2 Dry Blotting system (Thermo Fisher Scientific) at 20 V for 1 min, 23 V for 4 min and 25 V for 2 min. After transfer, membranes were soaked in deionised water (dH2O) before staining for total protein using the Revert™ 700 Total Protein Stain Kit according to manufacturer's instructions and proceeded to immunoblotting.

Immunoblotting was performed as follows. Membranes were blocked using Intercept^®^ TRIS buffered saline (TBS) Blocking Buffer for 1 h at RT and incubated with target primary antibody (see Table 1 for details) in TBS Blocking Buffer 0.1% Tween‐20 (Sigma‐Aldrich, Missouri) ON at 4°C, with mild shaking. Secondary antibody detection was performed after washing the primary antibody with 1X TBS (50 mM Tris‐Cl, pH 7.5, 150 mM NaCl (Sigma‐Aldrich)) with 0.1% Tween‐20. Primary antibody detection was performed using secondary IRDye^®^ 800CW in Intercept^®^ TBS Blocking Buffer 0.1% Tween‐20, for 1 h at RT. Membranes were washed again as before and imaged using the LI‐COR Odyssey ^®^ CLx Imaging System and quantified according to manufacturer's instructions using the Image Studio™ Lite Software (Li‐COR) and Microsoft Excel (Microsoft Corporation. (2018) v16.16.27) to process data.

### 5.11 Statistical analysis

Statistics were performed using the GraphPad Prism v7.00. Normality was assessed by the Shapiro–Wilk test. For normally distributed data where most of the groups had a sample size of ≥5, unpaired *t*‐test was used to compare 2 data points and one‐way ANOVA followed by Bonferroni post hoc test was used to compare more than 2 data points. For non‐normally distributed data and/or data containing less than 5 animals in most of the groups Mann–Whitney test and Kruskal–Wallis tests were used instead. Two‐way ANOVA followed by Tukey's multiple comparisons was used in order to compare more than 2 data points in 2 different groups (genotypes). Chi‐square was performed to analyse structure and morphological changes in the retina based on qualitative assessment, having taken into account the number of animals *per* group displaying defects versus not displaying defects. A critical value for significance of *p* < 0.05 was used throughout the analysis. There were no repeated measurements performed in this study.

## CONFLICT OF INTEREST

The authors declare no competing interests.

## AUTHOR CONTRIBUTIONS

RRM, CMH and RBM conceived, designed, performed, analysed and prepared Figures for the experiments relative to Figures 1, 2, 3, 4, 5 and Figures [Link], [Link], [Link], [Link], [Link], [Link]. MZ, MM and RT conceived, designed, performed, analysed and prepared experiments relative to Figure 6 and Figure S7. DTW performed the histopathology analysis. RRM, CMH and RBM wrote the manuscript, with input from MM and RT.

## Supporting information

Fig S1

Fig S2

Fig S3

Fig S4

Fig S5

Fig S6

Fig S7

Video S1

Video S2

Video S3

Video S4

## Data Availability

The datasets and source data generated during and/or analysed during the current study are available from the corresponding author upon reasonable request.
