# Supplementary figures and images for: Müller Glia maintain their regenerative potential despite degeneration in the aged zebrafish retina
Source: Aging Cell. 2022 Mar 22;21(4):e13597. doi: 10.1111/acel.13597 (PMC9009236; doi:10.1111/acel.13597)

**A**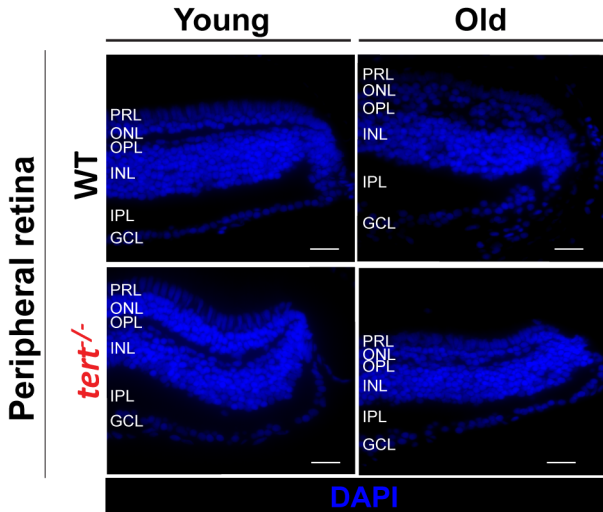**A'****Overall thickness in peripheral retina**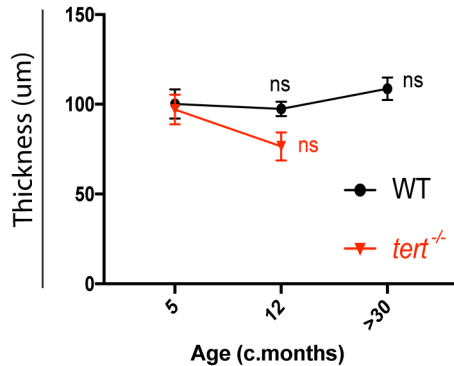

Supplement: Supplementary file 1 — Fig S1 [file ACEL-21-e13597-s003.pdf]

**Old**

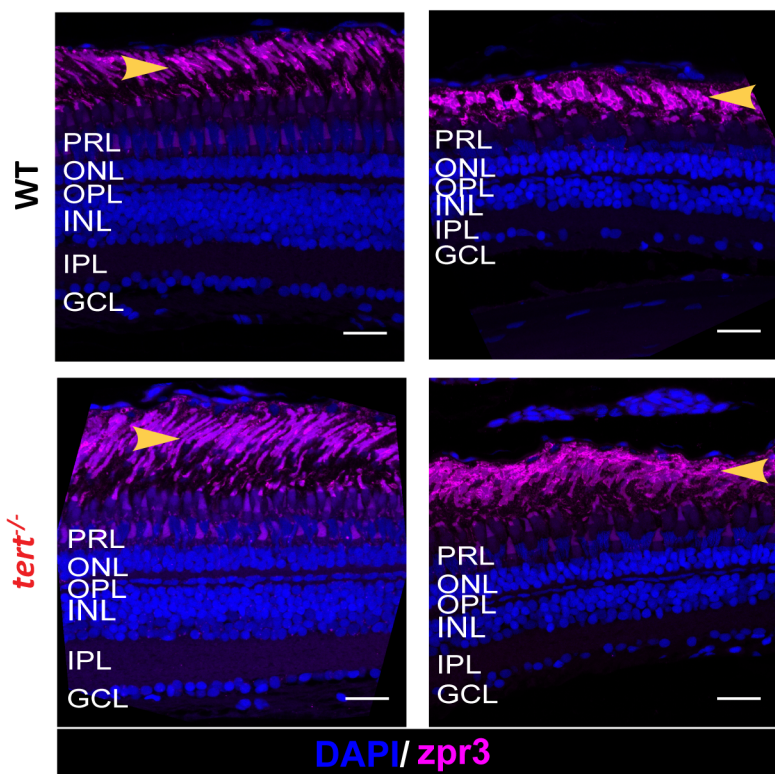

**A'**

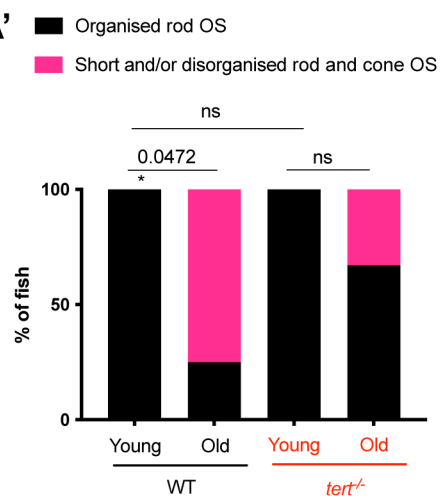

# B

## Young

**Old**

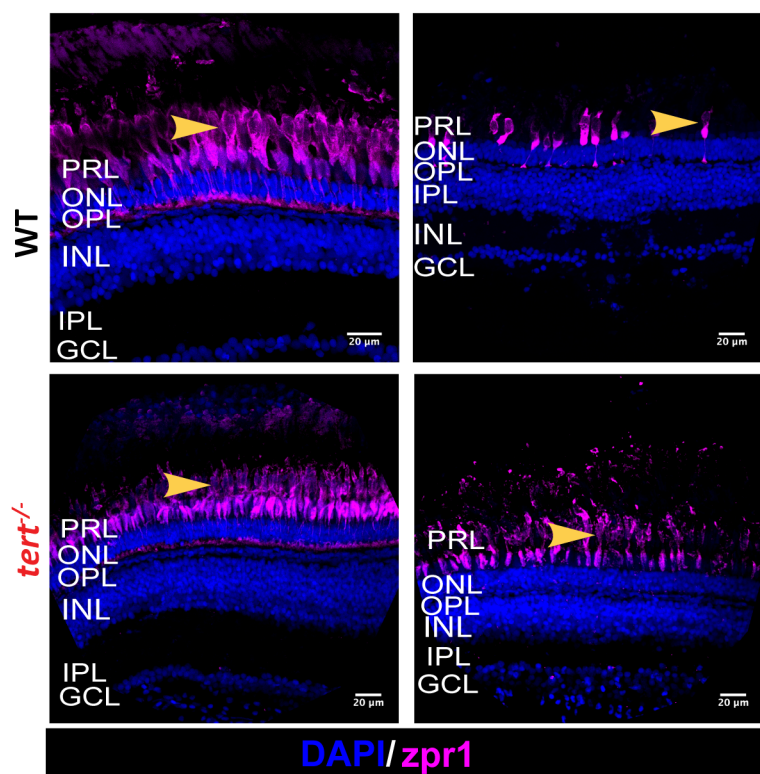

**B'**

### Red green cone length

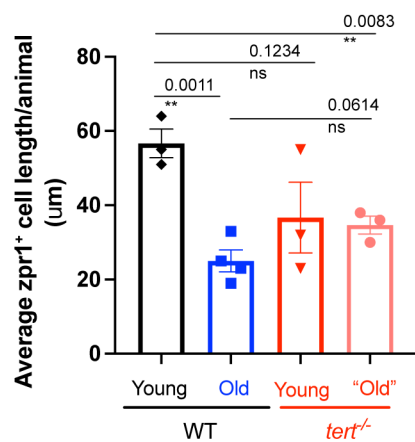

**B''**

### Red green cone numbers

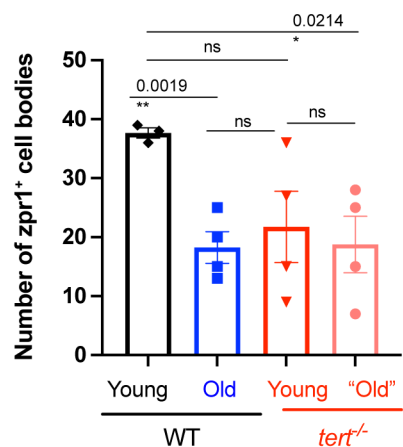

Supplement: Supplementary file 3 — Fig S3 [file ACEL-21-e13597-s002.pdf]

# Proliferation

A

Young

Old

WT

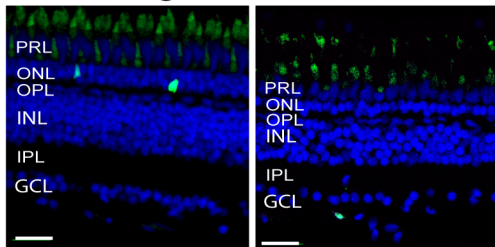

DAPI PCNA

A'

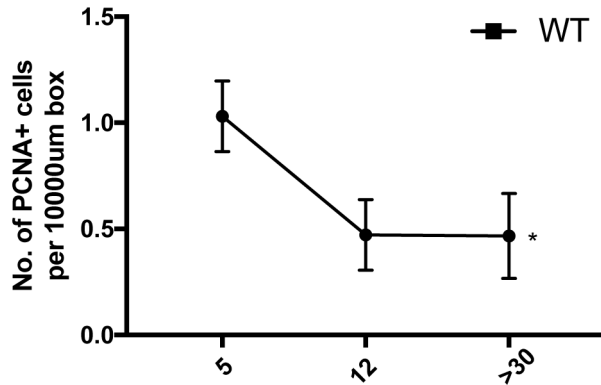

Supplement: Supplementary file 4 — Fig S4 [file ACEL-21-e13597-s007.pdf]

**A**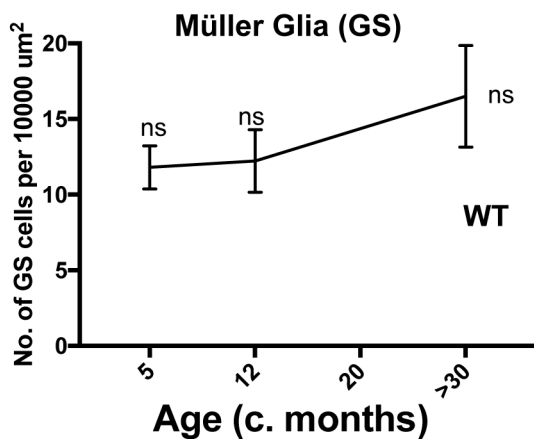**B****Microglia (4C4)**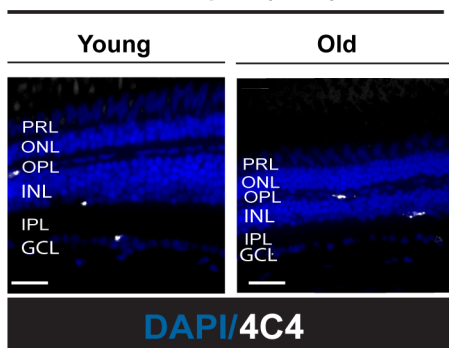**B'**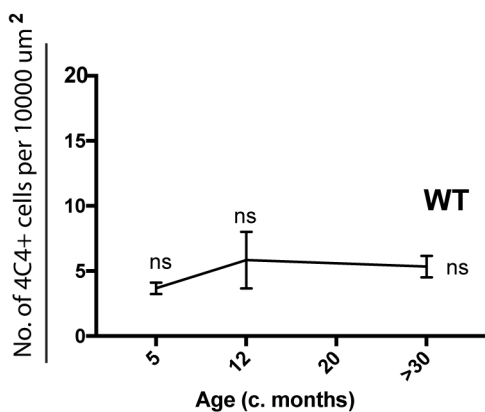**B''****Microglia numbers per individual WT retinal layer**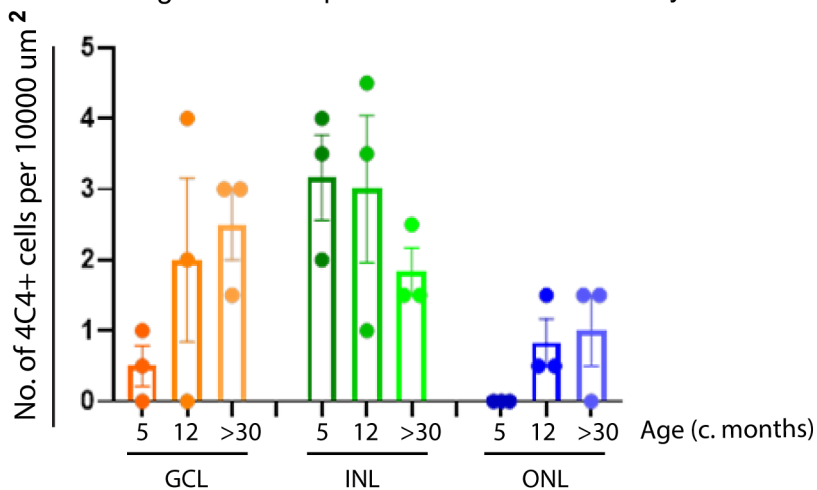

Supplement: Supplementary file 5 — Fig S5 [file ACEL-21-e13597-s006.pdf]

Whole protein stain  
(Revert™ 700 Total Protein Stain)

**A**

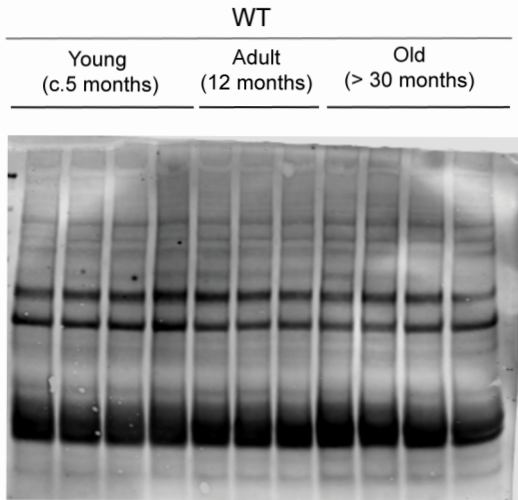

Supplement: Supplementary file 6 — Fig S6 [file ACEL-21-e13597-s009.pdf]

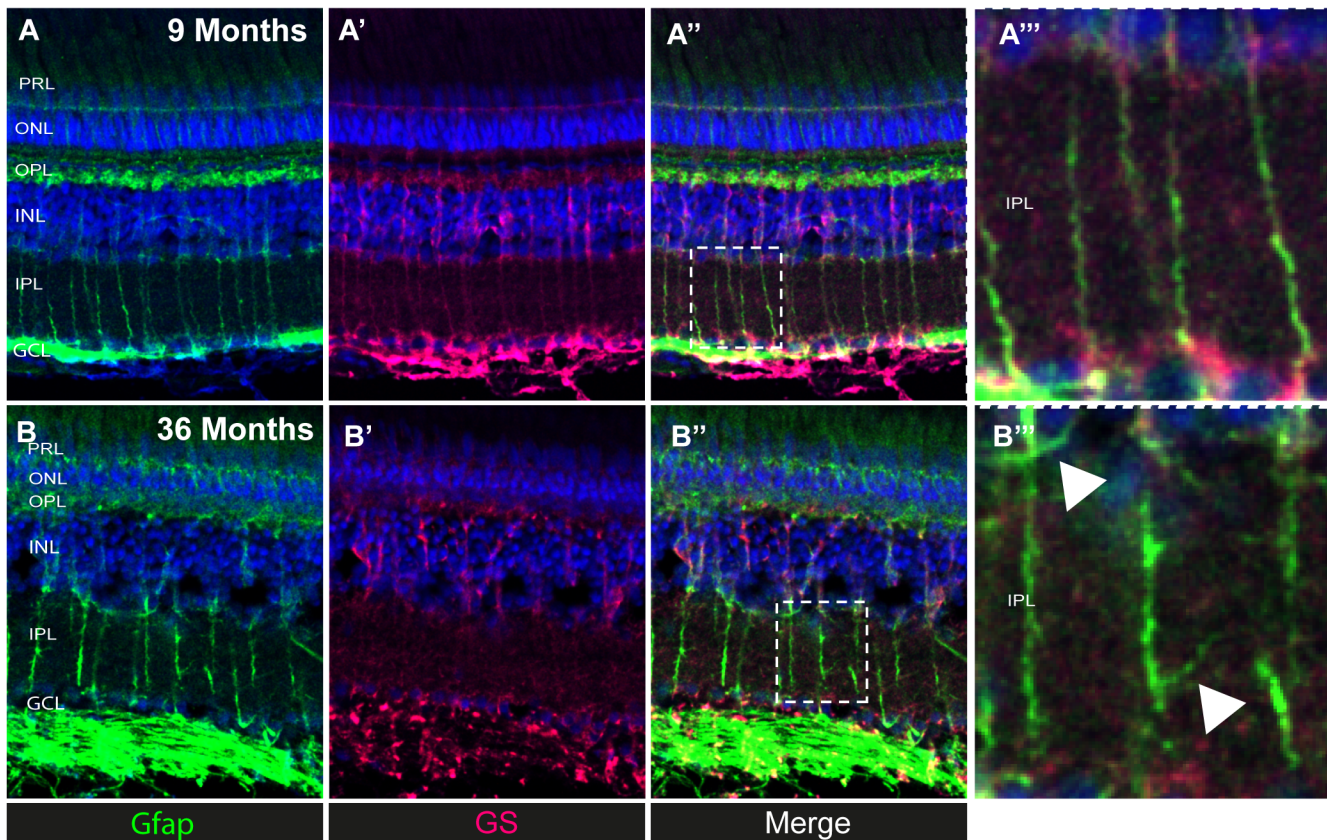

Supplement: Supplementary file 7 — Fig S7 [file ACEL-21-e13597-s001.pdf]
