# Supplementary material for: Müller Glia maintain their regenerative potential despite degeneration in the aged zebrafish retina
Source: Aging Cell. 2022 Mar 22;21(4):e13597. doi: 10.1111/acel.13597 (PMC9009236; doi:10.1111/acel.13597)

A

Phenotypes that score one point each	Cellular spatial arrangement unusual and/or cellular atrophy in INL	Cellular spatial arrangement unusual and/or cellular atrophy in PR layer	Thinning of the entire INL	Thinning of the entire PR layer	Unusual pigment "clumping" in RPE layer
Score 0 (a)	Normal architecture (or as close to normal) + thicker retinal layers overall (i.e associated with younger retinas)				
Score 0 (b)	Normal architecture (or as close to normal) + thinner retinal layers overall (i.e associated with older retinas but no other obvious pathology)				

B

	Cumulative score				
**Results**	**1**	**2**	**3**	**0 (a)**	**0 (b)**
**Samples (genotypes and ages)**	WT 9m<br>*tert*$^{-/-}$ 9m<br>WT 36m (wasted)<br>WT 36m (wasted)	*tert*$^{-/-}$ 9 m<br>WT 5m<br>*tert*$^{-/-}$ 11m (wasted)<br>WT 32m<br>WT 47m<br>WT 50m	*tert*$^{-/-}$ 5m<br>*tert*$^{-/-}$ 17m (wasted)<br>WT 15m<br>*tert*$^{-/-}$ 15m	WT 5m<br>WT 5m<br>*tert*$^{-/-}$ 5m<br>WT 32m<br>WT 4m<br>*tert*$^{-/-}$ 4m<br>*tert*$^{-/-}$ 12m	WT 5m<br>*tert*$^{-/-}$ 5m<br>WT 15m<br>WT 15m<br>*tert*$^{-/-}$ 16m (wasted)

B'

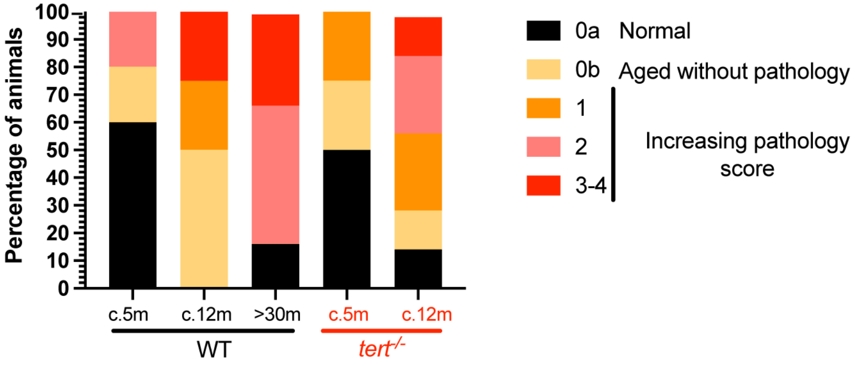

C

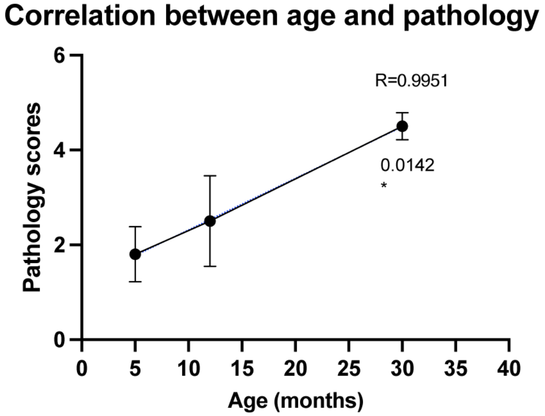

Supplement: Supplementary file 2 — Fig S2 [file ACEL-21-e13597-s004.pdf]
